# A suite of large language models for public health infoveillance

**DOI:** 10.1038/s41746-026-02435-6

**Published:** 2026-02-23

**Authors:** Xinyu Zhou, Jiaqi Zhou, Chiyu Wang, Qianqian Xie, Kaize Ding, Chengsheng Mao, Yuntian Liu, Zhiyuan Cao, Huangrui Chu, Xi Chen, Hua Xu, Heidi J. Larson, Yuan Luo

**Affiliations:** 1 Division of Biostatistics and Informatics, Department of Preventive Medicine, Northwestern University, Chicago, IL 60611 USA; 2 Health Science Integrated PhD Program, Feinberg School of Medicine, Northwestern University, Chicago, IL 60611 USA; 3 Department of Computer Science, Yale University, New Haven, CT 06511 USA; 4 Department of Biomedical Informatics & Data Science, Yale School of Medicine, New Haven, CT 06510 USA; 5 Department of Statistics and Data Science, Northwestern University, Evanston, IL 60208 USA; 6 Department of Biostatistics, Yale School of Public Health, New Haven, CT 06510 USA; 7 Department of Health Policy and Management, Yale School of Public Health, New Haven, CT 06510 USA; 8 Department of Economics, Yale University, New Haven, CT 06511 USA; 9 Department of Infectious Disease Dynamics, London School of Hygiene and Tropical Medicine, London, W1E 7HT UK; 10 Institute for Health Metrics and Evaluation, University of Washington, Seattle, WA 98195 USA; 11 Center for Collaborative AI in Healthcare, Institute for AI in Medicine, Feinberg School of Medicine, Northwestern University, Chicago, IL 60611 USA

**Keywords:** Health policy, Public health

## Abstract

Social media is a critical platform for understanding and fostering public engagement with health interventions. However, the lack of real-time social media infoveillance on public health issues may lead to delayed responses and suboptimal policy adjustments. To address this gap, we developed PH-LLM—a novel suite of large language models (LLMs) designed for real-time public health monitoring. We curated a multilingual training corpus and trained PH-LLM using QLoRA and LoRA plus, leveraging Qwen 2.5. We constructed a benchmark comprising 19 English and 20 multilingual held-out tasks and evaluated PH-LLM’s zero-shot performance. PH-LLM consistently outperformed baseline LLMs of similar and larger sizes. PH-LLM-14B and PH-LLM-32B surpassed Qwen2.5-72B-Instruct, Llama-3.1-70B-Instruct, Mistral-Large-Instruct-2407, and GPT-4o in both English tasks (>=56.0% vs. <= 52.3%) and multilingual tasks (>=59.6% vs. <= 59.1%). PH-LLM represents a significant advancement in real-time public health infoveillance, offering state-of-the-art multilingual capabilities and cost-effective solutions for monitoring public sentiment on health issues.

## Introduction

The effectiveness of public health interventions, such as social distancing, COVID-19 testing, and vaccination, hinges on collective support, participation, and adherence in both physical and virtual platforms. With recent advances in machine learning, infoveillance—the continuous analysis of online text information^1^—has emerged as a supplement to traditional public health surveillance approaches, offering early insights into public responses to interventions. Infoveillance has also been employed to mitigate the infodemic–an overwhelming surge of information and misinformation that may lead to deleterious public health consequences during pandemics, in addition to ensuring public adherence and informing health policy decisions^1–5^.

A growing number of public health researchers and authorities are leveraging social media data to explore vaccine attitudes, mental health issues, adherence to non-pharmaceutical interventions (NPIs), the spread of misinformation, and beyond, with machine learning models such as random forest and naïve bayes^2,6,7^. Despite these efforts, real-time infoveillance on social media platforms remains limited, especially when tracking rapidly evolving public health emergencies like COVID-19^6,7^. Without timely and scalable infoveillance methods, there may potentially be delays in policy refinement and missed opportunities for prompt public health interventions^7^.

Large Language Models (LLMs) hold promising potentials for infoveillance^8–13^. They can perform infoveillance tasks without necessitating the extensive resources, time, and task-specific annotated datasets typically required for training conventional machine learning models for large-scale infoveillance. Moreover, their human-like interactions make them more accessible to public health experts than many other machine learning tools. However, proprietary LLMs such as ChatGPT are associated with high cost and may lead to data leakage. On the other hand, open-source LLMs targeted at general tasks are not optimized for public health information surveillance. There’s a need for developing LLMs tailored for public health infoveillance, which could significantly reduce costs while delivering state-of-the-art performance.

In this study, we introduce PH-LLM (Public Health Large Language Models for infoveillance), which is a novel suite of LLMs specifically trained for multilingual infoveillance on social media platforms. We designed the first multilingual public health infoveillance benchmark, where we evaluated PH-LLM against leading open-source and proprietary LLMs, including GPT-4o. The PH-LLM models and associated Python code can be publicly accessible at https://github.com/luoyuanlab/PH-LLM.

## Results

Table 1 compares the zero-shot performance of PH-LLM on 19 tasks across six English-language datasets against other open-source LLMs of similar sizes (Supplementary Fig. 2). PH-LLM models demonstrated superior average performance, as measured by F_1_-score and micro F_1_-score, compared to their counterparts. Specifically, the smallest model, PH-LLM-0.5B, achieved an average model performance of 30.3%, outperforming Qwen2.5-0.5B-Instruct (23.6%) across 14 of 19 tasks. PH-LLM-1.5B achieved 39.9%, surpassing both Qwen2.5-1.5B-Instruct (36.3%) and the similar-sized Llama-3.2-1B-Instruct (28.0%) on 15 and 16 out of 19 tasks, respectively. Among models with ~7 billion parameters, PH-LLM-7B achieved 48.7%, outperforming bloomz-7b1-mt (27.9%) and Llama-3.1-8B-Instruct (45.4%). However, it performed slightly below Qwen2.5-7B-Instruct (50.7%). PH-LLM-14B (56.0%) consistently outperformed Qwen2.5-14B-Instruct (48.9%) across 13 out of 19 tasks and exceeded Mistral-Nemo-Instruct-2407 (47.1%) on 17 tasks. Remarkably, it also surpassed Mistral-Small-Instruct-2409 (45.8%), which has a larger parameter size of 22 billion. The largest model, PH-LLM-32B, achieved an average performance of 57.9%, surpassing Qwen2.5-32B-Instruct (52.5%).

**Table 1:** Comparison of zero-shot performance on English-language datasets between PH-LLM and other open-source LLMs of similar sizes

		PH-LLM-0.5B	Qwen2.5-0.5B-Instruct	PH-LLM-1.5B	Qwen2.5-1.5B-Instruct	Llama-3.2-1B-Instruct	PH-LLM-3B	Qwen2.5-3B-Instruct	Llama-3.2-3B-Instruct	PH-LLM-7B	Qwen2.5-7B-Instruct	bloomz-7b1-mt	Llama-3.1-8B-Instruct	PH-LLM-14B	Qwen2.5-14B-Instruct	Mistral-Nemo-Instruct-2407	Mistral-Small-Instruct-2409	PH-LLM-32B	Qwen2.5-32B-Instruct
	Model size	0.49B	0.49B	1.5B	1.5B	1.23B	3.1B	3.1B	3.21B	7.6B	7.6B	7.1B	8B	14.7B	14.7B	12B	22B	32.5B	32.5B
Dataset	Task																		
CAVES	A	***12.6***	9.3	***23.1***	19	12.5	25	***28.3***	10.9	28.4	***39***	13.8	21.6	***34.2***	26.1	22	0	***41.7***	30.5
	B	***18***	15.9	***24***	17.5	14.6	***23.4***	22.4	23.3	***35.2***	32.3	12.7	23.4	37.5	36.1	24	***43.5***	***43***	40
	C	***22.4***	21.4	***34.6***	29.2	22.8	***48.1***	42	31.1	41.4	***51.7***	20.6	42.8	***59.4***	41.3	47.6	6	***58.9***	55.9
	D	***13.6***	10.7	***15.4***	13.9	11.9	***22***	19.7	13.6	17.7	***24.2***	10.7	18.4	***25***	23.9	15.5	21.5	***28.8***	24.3
	E	***27.5***	25.7	***38.7***	29.9	26.2	42.5	***51.6***	37.4	40.4	***55.8***	25.3	44	44.1	55.9	41.5	***59.5***	***52.7***	51
	F	36.5	***51.2***	***64.1***	58.8	55.6	***70***	60.9	66.7	***72.1***	67.9	44.8	68.3	***77.2***	59.2	74.8	50.8	***78.5***	73.5
	G	21.5	***24.7***	***39.1***	32.8	29	40	***42.8***	35.4	41.2	***47.2***	24.3	35.5	54.5	***58.4***	39.9	42.2	***62.1***	54.8
CC	CC	***67.9***	30	79.4	***87.4***	57.5	79	***89.3***	89.1	***91.5***	87.8	52.2	78.8	***92.9***	88.4	90.3	88.5	***91.2***	86.8
Ethos		***57.5***	56.2	***71.3***	63.9	51.1	***77.7***	54	77.2	80	62.7	42.7	***81.1***	80.3	82.7	***85.2***	84.7	83.8	***85.9***
GHC	A	***23.9***	13.2	***36.5***	24.7	14.5	***43.8***	39.7	28.5	41.8	43.8	15.2	***45***	48.9	***50.1***	46.5	21.1	***48.5***	48
	B	***29.3***	10.8	***35.4***	16.4	5.5	***32.6***	23.3	26.5	39.9	***45***	11.4	32.4	***49***	47.6	43.4	44.2	48	***48.1***
MC	A	***39.6***	9.4	44.9	***50.1***	14.6	48.2	***48.3***	50	46.8	***51.9***	39.7	46.5	50	***56***	51.7	54.9	52.8	***53.2***
	B	***37.5***	35.2	***63.7***	62.5	29.4	62	***65.4***	57.9	67.2	***71.2***	26.6	70.2	***78.5***	73.8	69.1	72.2	***77.6***	77.1
	C	***22.1***	16.7	***42.7***	37.6	21.5	40.3	***42.1***	42.3	47.7	***58.7***	22.6	49	52.3	51.8	48	***67.9***	54.5	***55.4***
	D	***16.4***	***16.4***	***24.8***	23.3	13.4	26.8	***27.4***	24.3	28.3	35	21.5	***39.4***	44	***44.9***	43.2	35	38.5	***46.4***
	E	***5.4***	3	***16.4***	3	0	19.1	***20***	15.9	***21.3***	19.2	10.9	14.9	***30.5***	20.5	12	4.8	***24.2***	21.9
TCT	A	***79.4***	7.8	55.4	39.7	***71.7***	***63.5***	59.3	34.8	76	***89.4***	68.5	79.5	74.2	59.7	73.2	***90.5***	***84.3***	59.7
	B	41.9	***62.2***	40.7	51.4	***52.6***	***32***	20.2	34	***50.2***	26.1	40.3	24.3	***67***	8.8	12.2	29	***57.4***	29.8
	C	2.4	***27.8***	7.1	29.2	***27.7***	55.9	53.8	***27.9***	57.8	53.9	***25.9***	48.3	***64.4***	44.7	54.9	53.9	***74.2***	55.3
average		**30.3**	23.6	**39.9**	36.3	28.0	**44.8**	42.7	38.3	48.7	**50.7**	27.9	45.4	**56.0**	48.9	47.1	45.8	**57.9**	52.5

Descriptions of datasets and tasks presented: CAVES: A dataset concerning COVID-19 vaccine (Classification task A: vaccine not necessary, B: freedom, C: companies making money, D: distrust in policymakers, E: clinical trials were not reliable, F: side effects, G: distrust in effectiveness); CC: a dataset classifying personal narrative and news; Ethos: classifying hate speech; GHC: a hate speech dataset (Classification task A: assaults on human diginity, B: offensive language towards individuals); MC: a COVID-19 misinformation dataset (Classification task A: calling out or correction, B: conspiracy, C: politics, D: sarcasm or satire, E: false fact or prevention); TCT: a dataset on COVID-19 test: (Classification task A: tweets sent by individual users about COVID-19 test, B: supporting mass COVID-19 testing, C: mentioning COVID-19 test for certain subpopulations). Boldface and italics are used to mark the best-performing model for each task among models of similar size. Shaded cells highlight the performance of PH-LLM models. Underlining is used to emphasize the average performance within each model. 95% confidence intervals based on bootstrap sampling (*n* = 1000) are available in Supplementary Data.

Table 2 presents the zero-shot performance of PH-LLM models on 20 tasks across four multilingual datasets with the same set of benchmark LLMs, where PH-LLM consistently outperformed other models of similar sizes. PH-LLM-0.5B improved upon Qwen2.5-0.5B-Instruct (34.5% vs. 29.5%) on 17 out of 20 tasks. Similarly, PH-LLM-1.5B (42.1%) outperformed both Qwen2.5-1.5B-Instruct (34.1%) and Llama-3.2-1B-Instruct (27.7%), while PH-LLM-3B (48.1%) outperformed both Qwen2.5-3B-Instruct (41.1%) and Llama-3.2-3B-Instruct (40.0%). Among models with~7 billion parameters, PH-LLM-7B (58.5%) consistently outperformed blooms-7b1-mt (27.3%), as well as Qwen2.5-7B-Instruct (47.4%) and Llama-3.1-8B (47.2%) on most of the 20 tasks. PH-LLM-14B (59.6%) also surpassed Qwen2.5-14B-Instruct (51.5%), Mistral-Small-Instruct-2407 (42.9%), and Mistral-Small-Instruct-2409 (47.4%) in most tasks. PH-LLM-32B achieved an average performance of 61.4%, exceeding Qwen2.5-32B-Instruct’s 55.1%.

**Table 2:** Comparison of zero-shot performance on multilingual datasets between PH-LLM models and other open-source LLMs of similar sizes

		PH-LLM-0.5B	Qwen2.5-0.5B-Instruct	PH-LLM-1.5B	Qwen2.5-1.5B-Instruct	Llama-3.2-1B-Instruct	PH-LLM-3B	Qwen2.5-3B-Instruct	Llama-3.2-3B-Instruct	PH-LLM-7B	bloomz-7b1-mt	Qwen2.5-7B-Instruct	Llama-3.1-8B-Instruct	PH-LLM-14B	Qwen2.5-14B-Instruct	Mistral-Nemo-Instruct-2407	Mistral-Small-Instruct-2409	PH-LLM-32B	Qwen2.5-32B-Instruct
	Model size	0.49B	0.49B	1.5B	1.5B	1.23B	3.1B	3.1B	3.2B	7.6B	7.1B	7.6B	8B	14.7B	14.7B	12B	22B	32.5B	32.5B
Dataset	Task																		
AHSFN	A	***28.8***	1.3	***31.4***	30.5	14.4*	43.6	***44.2***	14.5*	***63.7***	10.1	21.5	22.6*	***66.7***	58	42.2*	8.3*	***49.5***	32.2
	B	16.5	***26.9***	***56.4***	41.6	27*	***70.3***	65.8	50.9*	***72.7***	29.7	69	64.6*	***79.9***	73.1	63.2*	67.9*	86.9	***90***
	C	26.8	***29.2***	30.9	***31.9***	24.6*	35.1	***44***	34.5*	61	25.3	***63.9***	48.6*	***59.4***	38.6	18.7*	37.2*	60.7	***68***
	D	***24.2***	22.8	26.4	***28***	20.7*	23.3	35.5	38.5*	28.4	20	***39***	24.3*	24.5	***40.7***	20.2*	40.1*	27.2	***37.1***
	E	***44.7***	34.4	***78.7***	64.8	46.2*	71.6	***79.5***	78.2*	83.6	28.9	***85.7***	81.8*	81.1	***87.1***	85.7*	85.9*	77.1	***83.5***
	F	***43.4***	43.2	***51.4***	52.6	52.1*	***56.4***	52.6	44*	62.5	46.2	58.5	65.9*	56.4	56.2	58.0*	63.2*	65.7	***76.9***
	G	***13.3***	11.2	***19.8***	13.5	11.8*	***23.4***	18.9	16.4*	30.4	11.8	18.7	30.6*	22.9	29.4	25.4*	30.8*	20.3	***28.7***
	H	***64.3***	57.6	***63.3***	55.3	43.1*	***75.2***	68.9	63.5*	***82.2***	28.4	68.2	78.1*	79.8	83.1	85.7*	64.1*	74.4	***81.2***
	I	***22.8***	8	***35.9***	5.1	24.1*	35.7	4.3	45.7*	***47.4***	33.8	16.9	45.3*	***38.3***	21.7	18.3*	37.6*	***32.4***	22
	J	***10.3***	4.4	3.1	0	14.9*	***18.4***	0	14.7*	31.7	12.8	***33.6***	23.6*	***41.5***	24.7	10.7*	26.5*	***41.8***	35.2
ITED		***29.3***	26.7	53.3	***59.4***	30.4*	57.6	***62***	55.1*	67.9	25.4	***72.7***	65.3*	71.8	***73.8***	69.5*	70.1*	***73.6***	73.1
MAT		***17.6***	9.1	***25.2***	9.1	23.3*	28	0	31.4*	***46***	23.8	37.7	28.5*	***47.8***	47.1	29.5*	44.1*	***57.7***	51.3
WCV	A	76.4	***76.5***	***75.2***	63.8	43.9*	***82.8***	77.6	53*	***86.7***	51	74.1	47.5*	***87.4***	71.5	41.9	49.3	***89.4***	77.2
	B	***73.7***	73.1	***67.5***	44.3	45.7*	***76***	13.6	48*	***82***	53.8	8.2	46.9*	***82.7***	37.4	21.6	22.4	***83.3***	8.7
	C	***35.9***	32	19.9	1.7	24.8*	***53.5***	38.5	35.6*	33.1	22.7	28.8	43.8*	35.5	30.8	***36.7***	32.9	54	***61.2***
	D	***40***	35.4	***51.4***	46.8	27.2*	51.8	***52.2***	42.6*	***60.3***	28.7	56.7	52.2*	***67.9***	59.3	41.2	64.5	***73.5***	53.3
	E	***28.9***	23.2	***40.7***	28.8	19.3*	35	41.6	43.6*	***68.7***	19.7	51.4	53.1*	***67.2***	40.8	42.3	59.1	***71.2***	59.7
	F	***32.5***	29.2	***38.9***	27.8	11.3*	***44.4***	42.5	34.6*	***63.3***	31.9	49.3	39.8*	***65.2***	52.9	48.3	51.7	***60.2***	58.4
	G	***37.8***	29.5	40	***46.6***	32*	34.7	33.7	36.2*	***58.6***	25.5	52.6	49.4*	***61.9***	54	57.3	55.7	***62.2***	58.3
	H	***22.4***	15.6	***33.3***	29.8	16.3*	44.3	***45.7***	19.1*	40.7	16.1	***41.1***	31.3*	***53.8***	49.4	41.7	36.9	***66.7***	45
average	**34.5**	29.5	**42.1**	34.1	27.7*	**48.1**	41.1	40.0*	**58.5**	27.3	47.4	47.2*	**59.6**	51.5	42.9*	47.4*	**61.4**	55.1	

Descriptions of datasets and tasks presented: AHSFN: an Arabic dataset regarding hate speech and misinformation regarding COVID-19 (Classification tasks A: hate speech, B: cure or vaccine mentions, C: advice, D: encouraging tweets, E: news vs. opinions, F: dialects, G: blame and negative speech, H: whether the tweet can be verified, I: worth fact-checking, J: contain fake information); ITED: an Indonesian emotion detection dataset (classifying (1) anger, (2) happy, (3) sadness, (4) fear, (5) love); MAT: an Arabic dataset regarding classifying misinformation; WCV: a Chinese dataset regarding COVID-19 vaccine sentiment (Classification task A: classifying Weibo posts from personal accounts, B: vaccine acceptance, C: vaccine refusal, D: vaccine is effective, E: vaccine is not effective, F: vaccine is important, G: risk perception, H: negative information and misinformation). * indicates that language included in the dataset are not officially supported by the model. Boldface and italics are used to mark the best-performing model for each task among models of similar size. Shaded cells highlight the performance of PH-LLM models. Underlining is used to emphasize the average performance within each model. 95% confidence intervals based on bootstrap sampling (*n* = 1000) are available in Supplementary Data.

Table 3 and Table 4 present further comparison of PH-LLM models with larger open-source models and proprietary LLMs for both English-language and multilingual datasets (Supplementary Fig. 2). For English-language comparison (Table 3) across 19 tasks, the largest PH-LLM model, PH-LLM-32B (57.9%), demonstrated not only competitive but superior overall performance to other larger open-source models, such as Qwen2.5-72B-Instruct (49.6%) and Llama-3.1-70B-Instruct (52.3%), and Mistral-Large-Instruct-2407 (51.8%). Furthermore, PH-LLM-32B achieved state-of-the-art performance, outperforming both proprietary LLMs (46.9% of GPT-4o mini and 50.7% of GPT-4o). In Table 4, for multilingual datasets, PH-LLM-32B continues to outperform all other state-of-the-art models, achieving an average model performance of 61.4% across 20 tasks, specifically Qwen2.5-72B-Instruct (58.5%), Llama-3.1-70B-Instruct (57.7%), Mistral-Large-Instruct-2407 (56.6%), as well as GPT-4o mini (54.1%) and GPT-4o (59.1%).

**Table 3:** Comparison of zero-shot performance on English-language datasets between PH-LLM-32B and larger open-source models, flagship open-source models, and proprietary LLMs

		PH-LLM-32B	Qwen2.5-72B-Instruct	Llama-3.1-70B-Instruct	Mistral-Large-Instruct-2407	GPT-4o mini	GPT-4o
	Model size	32.5B	72.7B	70B	123B		
Dataset	Task						
CAVES	A	***41.7***	25.1	29.1	32	28.6	27.1
	B	***43***	24.5	36.9	37.7	34.4	40.4
	C	***58.9***	34.1	47.2	54	47.8	49.3
	D	28.8	16.7	22.8	23.6	16.9	***29.8***
	E	***52.7***	40.7	51.2	51.4	36	47.8
	F	78.5	***78.9***	73.4	76	75.8	76.7
	G	***62.1***	60.6	45.1	54.7	35.9	48.3
CC	CC	***91.2***	90.3	90.8	90.6	83.8	89.9
Ethos		83.8	84.5	84.7	86.8	86.4	***88.8***
GHC	A	48.5	***49.8***	41.1	48.7	41.4	41.9
	B	***48***	44	34.9	44.2	34.9	33.3
MC	A	52.8	57	***61.5***	50.5	56.6	55.7
	B	77.6	76.3	77.6	78.5	79.1	***81.7***
	C	54.5	55.4	52.9	***64***	48.7	57.5
	D	38.5	40.7	56	***62***	49	55.5
	E	24.2	***25.1***	23.5	24.5	24.9	24.1
TCT	A	***84.3***	63	82.5	27.7	40.1	25.8
	B	***57.4***	21.1	31.8	15.2	36.2	42.3
	C	***74.2***	55	51.2	62.1	35.2	47.1
average		**57.9**	49.6	52.3	51.8	46.9	50.7

Descriptions of datasets and tasks presented: CAVES: A dataset concerning COVID-19 vaccine (Classification task A: vaccine not necessary, B: freedom, C: companies making money, D: distrust in policymakers, E: clinical trials were not reliable, F: side effects, G: distrust in effectiveness); CC: a dataset classifying personal narrative and news; Ethos: classifying hate speech; GHC: a hate speech dataset (Classification task A: assaults on human diginity, B: offensive language towards individuals); MC: a COVID-19 misinformation dataset (Classification task A: calling out or correction, B: conspiracy, C: politics, D: sarcasm or satire, E: false fact or prevention); TCT: a dataset on COVID-19 test: (Classification task A: tweets sent by individual users about COVID-19 test, B: supporting mass COVID-19 testing, C: mentioning COVID-19 test for certain subpopulations). Boldface and italics are used to mark the best-performing model for each task among models of similar size. Shaded cells highlight the performance of PH-LLM models. Underlining is used to emphasize the average performance within each model. 95% confidence intervals based on bootstrap sampling (*n* = 1000) are available in Supplementary Data.

**Table 4:** Comparison of zero-shot performance on multilingual datasets between PH-LLM-32B and larger open-source models, flagship open-source models, and proprietary LLMs

		PH-LLM-32B	Qwen2.5-72B-Instruct	Llama-3.1-70B-Instruct	Mistral-Large-Instruct-2407	GPT-4o mini	GPT-4o
	Model size	32.5B	72.7B	70B	123B		
Dataset	Task						
AHSFN	A	***49.5***	43.9	35.9*	28.6*	39.8	39.6
	B	86.9	88.5	87.4*	89.9*	87	***90.4***
	C	60.7	68.2	60.9*	67*	58	***70.6***
	D	27.2	34.5	35.9*	42.6*	35.6	33.8
	E	77.1	***85.1***	80.5*	83.4*	83.7	84.3
	F	65.7	***80.8***	75.4*	78.7*	59.7	79.9
	G	20.3	35.6	35.9*	34.2*	31.6	35.1
	H	74.4	88.2	91.5*	90*	88.6	90
	I	32.4	33.9	40*	37.5*	***40.7***	29.4
	J	41.8	***42.6***	35.2*	31*	22.2	22.9
ITED		73.6	76.2	74.9*	74.5*	77.2	***78.1***
MAT		***57.7***	56.7	37.2*	51.1*	39.6	52.1
WCV	A	***89.4***	81.9	84.1*	81.6	83	81.8
	B	***83.3***	8.2	44.7*	14.2	38.6	43.1
	C	54	63.4	65.3*	57	54.2	60.6
	D	***73.5***	59.2	61.8*	54.5	64.1	68.5
	E	***71.2***	63.2	61.8*	66.7	55.4	67.1
	F	60.2	***60.7***	45.8*	52.3	44.3	50.7
	G	***62.2***	57.3	59.1*	58.4	51.2	***62.2***
	H	***66.7***	42.3	40*	39.1	28.1	41.7
average		**61.4**	58.5	57.7*	56.6*	54.1	59.1

Descriptions of datasets and tasks presented: AHSFN: an Arabic dataset regarding hate speech and misinformation regarding COVID-19 (Classification tasks A: hate speech, B: cure or vaccine mentions, C: advice, D: encouraging tweets, E: news vs. opinions, F: dialects, G: blame and negative speech, H: whether the tweet can be verified, I: worth fact-checking, J: contain fake information); ITED: an Indonesian emotion detection dataset (classifying (1) anger, (2) happy, (3) sadness, (4) fear, (5) love); MAT: an Arabic dataset regarding classifying misinformation; WCV: a Chinese dataset regarding COVID-19 vaccine sentiment (Classification task A: classifying Weibo posts from personal accounts, B: vaccine acceptance, C: vaccine refusal, D: vaccine is effective, E: vaccine is not effective, F: vaccine is important, G: risk perception, H: negative information and misinformation). * indicates that language(s) included in the dataset are not officially supported by the model. Boldface and italics are used to mark the best-performing model for each task among models of similar size. Shaded cells highlight the performance of PH-LLM models. Underlining is used to emphasize the average performance within each model. 95% confidence intervals based on bootstrap sampling (*n* = 1000) are available in Supplementary Data.

**Fig. 1:**
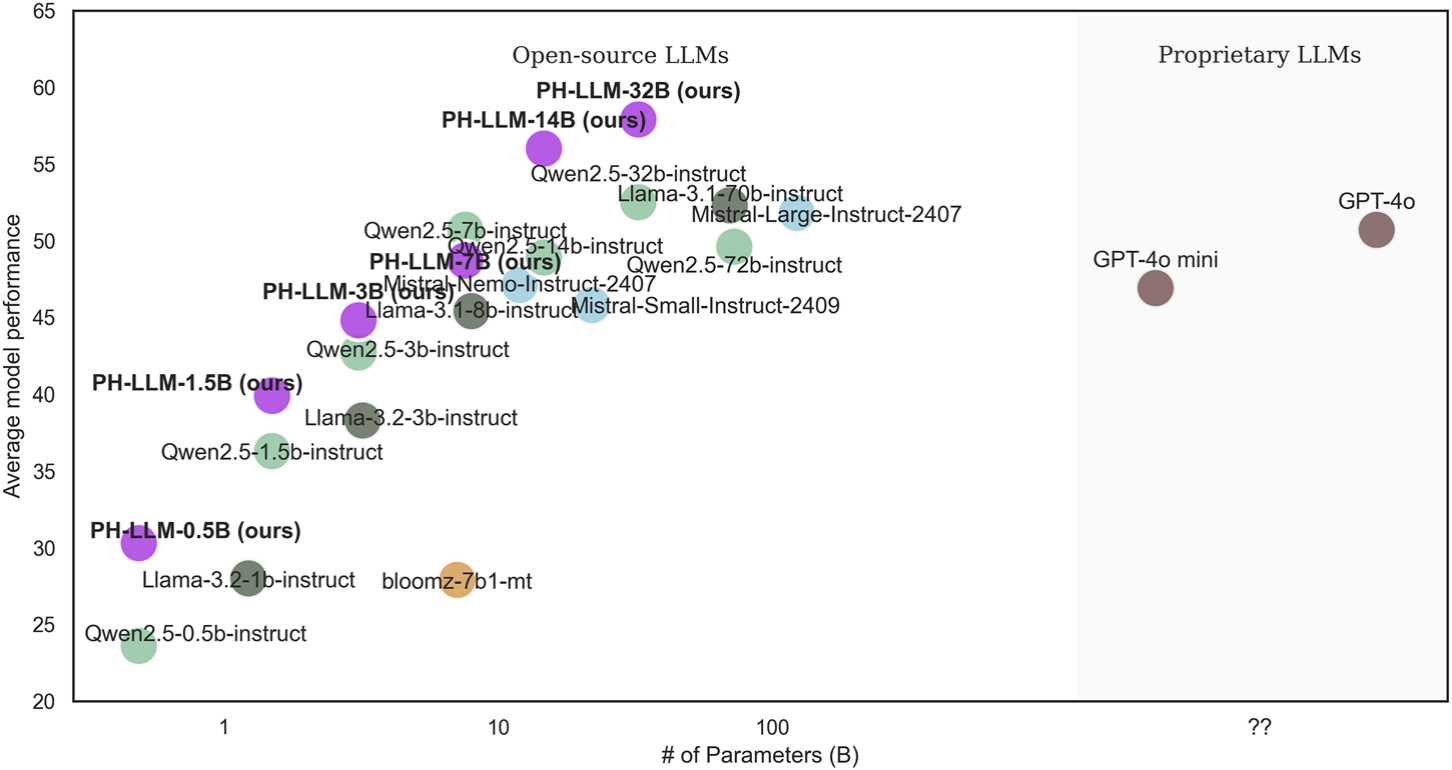
Relationship between model size and average zero-shot performance of LLMs in English evaluation datasets. Each point represents a model, plotted by parameter size (x axis) and average performance (y axis).

Figure 1 shows the relationship between average model performance and model size of LLMs evaluated across 19 English evaluation tasks. A positive relationship was observed between the number of parameters in open-source models and their performance. Notably, PH-LLM models demonstrated superior performance compared to models of similar sizes and even larger counterparts. PH-LLM-14B (56.0%) and PH-LLM-32B (57.9%) outperformed strong baselines, including GPT-4o (50.7%), Mistral-Large-Instruct-2407 (51.8%), and Llama-3.1-70B-Instruct (52.3%).

**Fig. 2:**
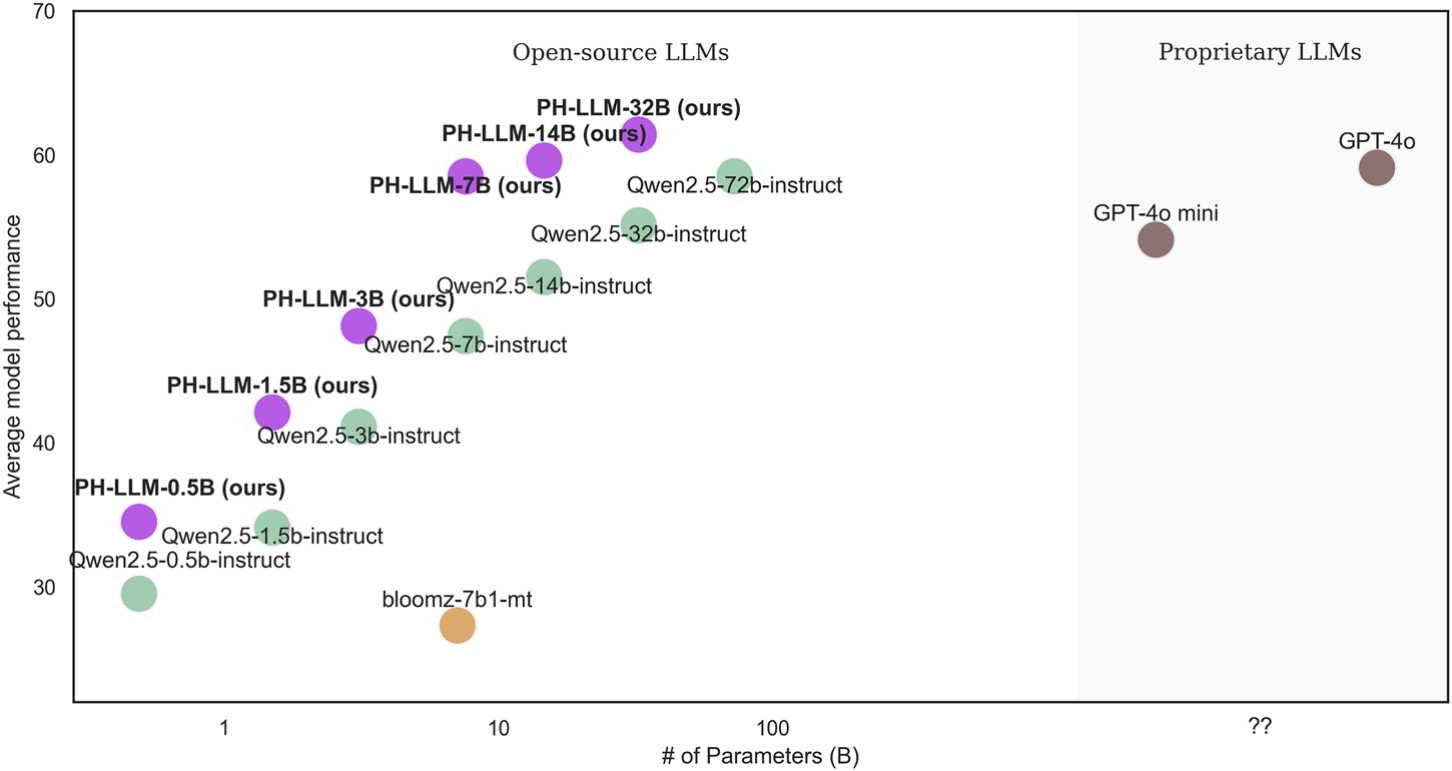
Relationship between model size and average zero-shot performance of LLMs in multilingual evaluation datasets for multilingual LLMs officially supporting all languages in the multilingual evaluation. Each point represents a model, plotted by parameter size (x axis) and average performance (y axis).

Figure 2 shows the relationship between average model performance and model size across 20 multilingual evaluation tasks. PH-LLM consistently outperformed models of similar sizes and, in some cases, larger models. PH-LLM-7B (58.5%), in particular, matched the average performance as Qwen2.5-72B-Instruct (58.5%). Moreover, both PH-LLM-14B (59.6%) and PH-LLM-32B (61.4%) surpassed state-of-the-art baseline models, including GPT-4o (59.1%), Qwen2.5-72B-Instruct (58.5%), and GPT-4o mini (54.1%).

PH-LLM models also demonstrated strong performance in 2-shot settings. (Supplementary Table 3, Supplementary Table 4).

## Discussion

In this study, we introduced PH-LLM, a novel suite of LLMs specialized in public health infoveillance. PH-LLM is available in six model sizes: PH-LLM-0.5B, PH-LLM-1.5B, PH-LLM-3B, PH-LLM-7B, PH-LLM-14B, and PH-LLM-32B. Across diverse public health infoveillance tasks, PH-LLM models consistently demonstrated strong performance, outperforming baseline models of comparable or larger sizes in most scenarios. Notably, PH-LLM-14B and PH-LLM-32B achieved superior overall performance on 39 tasks from 10 held-out datasets in public health infoveillance settings, surpassing all baseline models, including Llama-3.1-72b-instruct, Mistral-Large-Instruct-2407, Qwen2.5-72b-instruct, and GPT-4o.

PH-LLM can reach higher zero-shot performance in public health infoveillance tasks with a smaller number of parameters. It reduces the need for extensive GPU resources and complex infrastructure during model deployment and inference, lowering operational costs and making public health infoveillance more accessible, particularly for resource-constrained settings. PH-LLM’s adaptability enables localized and contextualized responses to diverse public health challenges, offering transformative potential for LMICs and other underserved regions.

Recent years have also seen the development of domain-specific LLMs in health, such as Med-PaLM and PMC-LLaMA^14,15^. However, these models are designed for clinical tasks rather than public health infoveillance. To the best of our knowledge, PH-LLM is the first suite of LLMs specialized in public health infoveillance, which is multilingual and publicly available. Previous studies have utilized general-purpose LLMs to advance public health infoveillance on social media platforms, including tasks like data augmentation in social media datasets^9,16^, and analyzing public health topics such as vaccine sentiment, mask-wearing behaviors, and mental health^8,10–13,17^. LLMs have also shown potential in assisting public health practice beyond infoveillance, including pandemic forecasting and information extraction^18,19^. However, almost all these studies applied general-purpose LLMs like LLaMA and ChatGPT rather than developing LLMs tailored for public health settings^20,21^, and they focused predominantly on English-language scenarios. PH-LLM emphasizes multilingual capabilities, extending its utility to non-English contexts, which addresses the diverse linguistic needs of global public health.

PH-LLM is designed to be accessible to public health professionals without requiring a background in computer science. With metadata (time, location, social-economic status, and beyond) associated with each social media post, aggregating predictions from PH-LLM can reveal spatiotemporal trends of opinions and behaviors, from nuances on social media platforms, and subsequently underline their public health significance. For example, to inform an HPV vaccination program, public health agencies can apply PH-LLM to stay updated with sudden changes in vaccine acceptance and confidence, trending concerns and misinformation on vaccines, and potential distrust in public health professionals, pharmaceutical companies, or the government. Additionally, tools like LlamaFactory enable users to interact with PH-LLM and effortlessly analyze large-scale data through a user-friendly interface^22^. PH-LLM exhibited strong zero-shot performance for analyzing social media posts relevant to public health. Its performance could be further enhanced potentially through prompt engineering and integration with retrieval-augmented generation and knowledge graph—incorporating contextualized and localized knowledge from public health experts.

PH-LLM equips public health systems with a tool to address future emerging infectious diseases and global health challenges. PH-LLM was trained and evaluated using datasets surrounding vaccine hesitancy, mental health, nonadherence to NPIs, hate speech, and misinformation, and similar challenges may re-emerge in future outbreaks and pandemics^23^. The generalizability of LLMs also allows PH-LLM to address new and evolving infoveillance topics with greater flexibility towards variations in geographies, languages, populations, and cultural, social, economic, and political contexts, which is an advantage over the pretrain-finetune paradigm. We mitigated potential biases during data collection by incorporating multilingual and cross-platform social media sources covering diverse populations and cultural contexts, then we further ensured fairness during evaluation by assessing model performance across multiple languages and tasks. However, we acknowledge that social media users represent a non-random subset of the population, potentially underrepresenting older adults and those with lower digital literacy. Future work should evaluate model performance across demographic subgroups where such metadata is available. Additionally, inherent biases in source annotations may propagate to model outputs, warranting continued research into fair and equitable infoveillance systems.

This study has several limitations. First, every LLM, including PH-LLM, demonstrated suboptimal results in specific tasks. This is because most of the evaluation tasks are imbalanced and could be challenging. Also, we did not optimize prompt templates to ensure fair comparisons and avoid overfitting. Task-specific prompt engineering and evaluations are recommended before deployment of LLMs in zero-shot public health infoveillance. Second, the training set included only 96 infoveillance tasks, which may limit the performance of PH-LLM on tasks less represented within the training corpus. Third, PH-LLM’s training datasets were derived from various previous studies, which may reflect inconsistency in annotation quality and potential biases introduced by annotators. Forth, social media data, which underpins PH-LLM’s training and evaluation, represents a biased subset of the population. Predictions based on such data should be interpreted with caution, especially in contexts involving censorship or self-censorship.

Despite these limitations, PH-LLM represents a significant enhancement as a novel suite of LLM tailored for public health infoveillance. Its public availability and state-of-the-art performance demonstrate its potential in public health monitoring and evidence-based policymaking, including in LMICs and among at-risk populations. PH-LLM aspires to equip public health agencies at all levels—global, national and local—with the power of AI to promote public health awareness, inform policy and interventions, and address future global health challenges.

## Methods

**Fig. 3:**
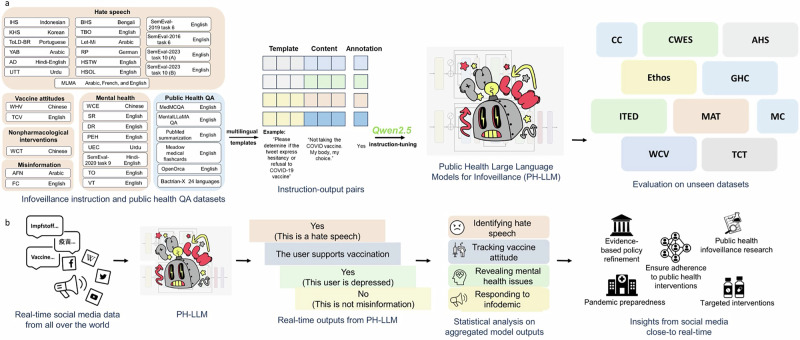
Overview of this study. **a** Construction and evaluation of PH-LLM, including collecting multilingual datasets, template-based instruction–output pairs generation, instruction tuning, and evaluation on unseen datasets. **b** Workflow of real-time infoveillance using PH-LLM. Social media data are processed by the model to generate outputs for infoveillance tasks, such as hate speech detection, vaccine attitude tracking, mental health signals, and misinformation identification, followed by statistical analysis to inform public health decision-making.

In this study, we developed a suite of LLMs named PH-LLM, available in six sizes for various computing settings: PH-LLM-0.5B, PH-LLM-1.5B, PH-LLM-3B, PH-LLM-7B, PH-LLM-14B, and PH-LLM-32B. These PH-LLM models were instruction-based fine-tuned on top of Qwen 2.5^24^, using a curated dataset of 593,100 instruction-output pairs based on 30 infoveillance datasets with a total of 96 public health infoveillance tasks and six question-answering datasets. We evaluated the PH-LLM models on 39 tasks with a total of 52,158 instruction-output pairs, across 10 datasets in English, Chinese, Arabic, and Indonesian. Notably, the evaluation datasets were distinct from those used during the development of PH-LLM. The performance of PH-LLM was benchmarked against state-of-the-art instruction-tuned LLMs, including GPT-4o (version 2024-05-13), Llama-3.1-70B-Instruct, Mistral-Large-Instruct-2407, and Qwen2.5-72B-Instruct^24–27^. An overview of this study is provided in Fig. 3.

### Data source

We conducted a Google search to compile an initial list of publicly available, manually annotated infoveillance datasets based on social media data. Two researchers (XZ and JZ, or XZ and CW) assessed the annotation quality of each dataset. The evaluation criteria included subjective impressions of the study’s quality, the robustness of the annotation process described, and the popularity of the paper and dataset as indicated by metrics such as citations and GitHub stars. Discrepancies between researchers were resolved through discussions. Only datasets that were manually annotated and deemed high quality by both researchers were recruited, either as the training set or the evaluation dataset. We prioritized datasets requiring API access for inclusion in the evaluation set. Datasets annotated using machine learning models were excluded. Ultimately, a total of 40 infoveillance datasets were collected. Of these, 30 datasets, encompassing 96 public health infoveillance tasks concerning vaccine sentiment, hate speech, mental health, NPIs, misinformation, and beyond, were included in the training set. In addition, six additional QA datasets were incorporated to enrich the training corpus. Details of the training set are provided in Supplementary Table 1. The remaining 10 datasets, comprising 39 unseen infoveillance tasks and 52,158 instruction-output pairs, were excluded from model training and reserved for evaluation. Details of the evaluation datasets are presented in Table 5. Importantly, there was no overlap between the training and evaluation sets.

**Table 5:** Evaluation benchmark

Data	Task	Language	Task description	Prompt template	Distribution of labels	Evaluation metric
English datasets						
CAVES (A Dataset to facilitate Explainable Classification and Summarization of Concerns towards COVID Vaccines)^38^	A	English	Vaccine not necessary	Please determine if the tweet below indicates COVID is not dangerous, vaccines are unnecessary, or that alternate cures (such as hydroxychloroquine) are better. \n If so, respond with ‘yes’. Otherwise, respond with ‘no’.\n Do not explain your rationale. Please directly respond with ‘yes’ or ‘no’.\n Tweet: [INSERT DATA HERE]\n Please respond with ‘yes’ or ‘no’.	145/1832	F_1_-score
	B		Freedom	Please determine if the tweet below is against mandatory vaccination and talks about their freedom\n If so, respond with ‘yes’. Otherwise, respond with ‘no’.\n Do not explain your rationale. Please directly respond with ‘yes’ or ‘no’.\n Tweet: [INSERT DATA HERE]\n Please respond with ‘yes’ or ‘no’.	157/1820	F_1_-score
	C		Companies making money	Please determine if the tweet below indicates that the Big Pharmaceutical companies are just trying to earn money, or is against such companies in general because of their history\n If so, respond with ‘yes’. Otherwise, respond with ‘no’.\n Do not explain your rationale. Please directly respond with ‘yes’ or ‘no’.\n Tweet: [INSERT DATA HERE]\n Please respond with ‘yes’ or ‘no’.	255/1722	F_1_-score
	D		Distrust in policymakers	Please determine if the tweet below expresses concerns that the governments / politicians are pushing their own agenda though the vaccines\n If so, respond with ‘yes’. Otherwise, respond with ‘no’.\n Do not explain your rationale. Please directly respond with ‘yes’ or ‘no’.\n Tweet: [INSERT DATA HERE]\n Please respond with ‘yes’ or ‘no’.	125/1852	F_1_-score
	E		Clinical trials were not reliable	Please determine if the tweet below expresses concerns that the vaccines have not been tested properly, have been rushed or that the published data is not accurate\n If so, respond with ‘yes’. Otherwise, respond with ‘no’.\n Do not explain your rationale. Please directly respond with ‘yes’ or ‘no’.\n Tweet: [INSERT DATA HERE]\n Please respond with ‘yes’ or ‘no’.	295/1682	F_1_-score
	F		Side effects	Please determine if the tweet below expresses concerns about the side effects of the vaccines, including deaths caused.\n If so, respond with ‘yes’. Otherwise, respond with ‘no’.\n Do not explain your rationale. Please directly respond with ‘yes’ or ‘no’.\n Tweet: [INSERT DATA HERE]\n Please respond with ‘yes’ or ‘no’.	762/1215	F_1_-score
	G		Distrust in effectiveness	Please determine if the tweet below expresses concerns that the vaccines are ineffective, not effective enough, or are useless.\n If so, respond with ‘yes’. Otherwise, respond with ‘no’.\n Do not explain your rationale. Please directly respond with ‘yes’ or ‘no’.\n Tweet: [INSERT DATA HERE]\n Please respond with ‘yes’ or ‘no’.	334/1643	F_1_-score
CC (COVID category)^39^		English	Personal narrative vs. news	Please categorize a given tweet text into either being a personal narrative or news.\n Tweet: “[INSERT DATA HERE]”. Now the tweet ends.\n Please respond with “news” or “personal”.	211/493	Micro F_1_
Ethos^40^		English	Hate speech	Please classify if the following social media post contain hate speech: [INSERT DATA HERE]. Now the post ends. Hate speech is a form of insulting public speech directed at specific individuals or groups of people on the basis of characteristics, such as race, religion, ethnic origin, national origin, sex, disability, sexual orientation, or gender identity. Please response with “Yes” if the post contains hate speech, and “No” if it does not.	84/116	F_1_-score
GHC (Gab Hate Corpus)^41^	A	English	Assaults on human dignity	Please determine if the following Gab post should be classified as assaults on human dignity (HD) or not: [INSERT DATA HERE]. Now the post ends. If so, respond with ‘yes’, otherwise respond with ‘no’. A document should be labeled as assaults on human dignity if it assaults the dignity of group by: asserting or implying the inferiority of a given group by virtue of intelligence, genetics, or other human capacity or quality; degrading a group, by comparison to subhuman entity or the use of hateful slurs in a manner intended to cause harm; the incitement of hatred through the use of a harmful group stereotype, historical or political reference, or by some other contextual means, where the intent of the speaker can be confidently assessed. In the evaluation of slurs against group identity (race, ethnicity, religion, nationality, ideology, gender, sexual orientation, etc.), we define such instances as hate-based if they are used in a manner intended to wound; this naturally excludes the casual or colloquial use of hate slurs. As an example, the adaptation of the N-slur (replacing the \-er” with \-a”) often implies colloquial usage. Language which dehumanizes targeted persons/groups will also be labeled as HD. In coding dehumanizing rhetoric, we refer coders to Haslam (2006), who developed a model for two forms of dehumanization. In mechanistic forms, humans are denied characteristics that are uniquely human (p. 252). Depriving the other from such traits is considered downward, animalistic comparison. Put another way, the target has been denied the traits that would separate them from animals. In another form of dehumanization as categorized by Haslam (2006), the target may be denied qualities related to human nature. These characteristics are traits that may not be unique to humans, but define them. These traits will represent the concept’s core but may not the same ones that distinguish us from other species” (p. 256). When these traits are denied from the target, this is considered upward, mechanistic dehumanization. The result of denial is often perceiving the target as cold, robotic, and lacking deep-seated core values and characteristics. Documents which invoke cultural, political, or historical context in order to voice negative sentiment/degradation toward a particular sub-population, empower hateful ideology (hate groups), or reduce the power of marginalized groups, are to be considered HD as well. This would include messages which indicate support for white supremacy (e.g. advocating for segregated societies/apartheid), those which make negative assertions and/or implications about the rights of certain groups (e.g. Immigrants in this country need to go back to their country), and those that reduce the power/agency of particular segments of the population. Now, please provide a response of either ‘yes’ or ‘no’ to the question above. You don’t need to provide any explanation.	491/5019	F_1_-score
	B		Offensive language towards individuals	Please determine if the following Gab post should be classified as vulgarity/offensive language directed at an individual (VO) or not: [INSERT DATA HERE]. Now the post ends. If so, respond with ‘yes’, otherwise respond with ‘no’. You don’t need to provide any explanation.	369/5141	F_1_-score
MC (Misinformation during COVID-19)^42^	A	English	Calling out or correction	Please determine if the tweet below meets any of the following conditions. If so, respond with “yes”, otherwise respond with “no”. The conditions are: 1. The tweet calls out or makes fun of a fake cure, a fake prevention, fake treatment, or a conspiracy theory. 2. The tweet links out to a site that debunks, calls out or makes fun of a fake cure, a fake prevention, fake treatment, or a conspiracy theory. 3. The tweet calls out or make fun of violations of social distancing rules or public health responses. 4. The tweet reports/quotes a (news) story related to consequences of a false fact, fake prevention, fake cure, fake treatment, or conspiracy theory. 5. The tweet reports/quotes a (news) story debunking a false fact, fake prevention, fake cure, fake treatment, or conspiracy theory. Please answer with “yes” or “no”. You don’t need to provide any explanation. Tweet: [INSERT DATA HERE]. Now the Tweet ends.	193/418	F_1_-score
	B		Conspiracy	Please determine if the tweet should be classified as conspiracy. If so, respond with “yes”, otherwise respond with “no”. A tweet shall be classified as a conspiracy if it endorses a conspiracy story. Some examples of conspiracy themes related to COVID-19 include: 1. It is a bioweapon. 2. Electromagnetic fields and the introduction of 5 G wireless technologies led to COVID-19 outbreaks. 3. This was a plan from Gates Foundation to increase the Gates’ wealth. 4. It leaked from the Wuhan Labs or Wuhan Institute of Virology in China. 5. It was predicted by Dean Koontz. Tweet: [INSERT DATA HERE]. Now the Tweet ends. Please answer with “yes” or “no”. You don’t need to provide any explanation.	100/511	F_1_-score
	C		Politics	Please determine if the tweet should be classified as politics. If so, respond with “yes”, otherwise respond with “no”. A tweet shall be classified as politics if the tweet mentions a political individual, institution, or government organization (eg. Congress, Democratic or Republican party), and any of the following conditions are met: 1. The tweet implicitly comments on actions taken by the political actor. 2. The tweet provides commentary on actions taken by the political actor. Tweet: [INSERT DATA HERE]. Now the Tweet ends. Please answer with “yes” or “no”. You don’t need to provide any explanation.	77/534	F_1_-score
	D		Sarcasm or satire	Please determine if the tweet below meets any of the following conditions. If so, respond with “yes”, otherwise respond with “no”. The conditions are: 1. The tweet contains clear signs of a satire calling out a fake cure, a fake prevention or a conspiracy. 2. The tweet includes a clear joke about a fake cure, a fake prevention or a conspiracy. Concretely, this is a tweet where the information in the post is false but is presented using humor, irony, exaggeration, or ridicule to expose and criticize people’s stupidity or vices, particularly in the context of contemporary politics and other topical issues. This kind of post is used to ridicule other false statements or people. Tweet: [INSERT DATA HERE]. Now the Tweet ends. Please answer with “yes” or “no”. You don’t need to provide any explanation.	76/535	F_1_-score
	E		False fact or prevention	Please determine if the tweet below meets any of the following conditions. If so, respond with “yes”, otherwise respond with “no”. The conditions are: 1. The tweet mention a false fact or prevention against COVID-19 that cannot be verified by the World Health Organization (WHO) or the Centers for Disease Control and Prevention (CDC). 2. The tweet mention a false fact or prevention against COVID-19 that is not supported by a peer-reviewed scientific study, or a preprint from reputable academic sources. Tweet: [INSERT DATA HERE]. Now the Tweet ends. Please answer with “yes” or “no”. You don’t need to provide any explanation.	52/559	F_1_-score
TCT (Twitter COVID test)^43^	A	English	Tweets sent by individual users about COVID-19 test	Please read a tweet and follow a data labeling request below.\n Tweet: [INSERT DATA HERE].\n Data labelling request: Please tell me if this is a tweet that is either a news report, sent by the government or government officials, sent by companies, advertisement, sent by bot, sent by any other non-personal accounts, retweet (RT/QT) from others without adding personal comments, or not related to coronavirus testing at all.\n If so, answer ‘yes’; otherwise, respond with ‘no’.	417/88	F_1_-score
	B		Supporting mass COVID-19 testing	Please read a tweet and follow a data labelling request below.\n Tweet: [INSERT DATA HERE].\n Data labelling request: Please tell me if this is a tweet that expresses understanding, supporting, accepting mass COVID-19 testing.\n If so, answer ‘yes’; otherwise, respond with ‘no’.	194/223	F_1_-score
	C		Mentioning COVID-19 test for certain subpopulations	Please read a tweet and follow a data labelling request below.\n Tweet: [INSERT DATA HERE].\n Data labelling request: Please tell me if this is a tweet that mentions COVID-19 test for certain subpopulations.\n If so, answer ‘yes’; otherwise, respond with ‘no’.	67/350	F_1_-score
Multilingual datasets						
AHSFN (Arabic Hate Speech and Fake News dataset regarding COVID-19)^44^	A	Arabic data and English prompt templates	Hate speech	Please determine if the provided tweet below contains hate speech. If so, respond “yes”. If not, respond “no”.\n. Tweet: “[INSERT DATA HERE]”. Now the tweet ends.\n. Please respond with ‘yes’ or ‘no’.	137/1602	F_1_-score
	B		Cure or vaccine mentions	Please determine if the provided tweet below contains any information or discussion about a cure, a vaccine, or other possible COVID-19 treatments. If so, respond “yes”. If not, respond “no”.\n. Tweet: “[INSERT DATA HERE]”. Now the tweet ends.\n. Please respond with ‘yes’ or ‘no’.	272/1468	F_1_-score
	C		Advice	Please determine if the provided tweet below tries to advise people or government institutions. If so, respond “yes”. If not, respond “no”.\n Tweet: “[INSERT DATA HERE]”. Now the tweet ends.\n Please respond with ‘yes’ or ‘no’.	276/1464	F_1_-score
	D		Encouraging tweets	Please determine if the provided tweet below contains encouraging, helpful, and positive speech. If so, respond “yes”. If not, respond “no”.\n Tweet: “[INSERT DATA HERE]”. Now the tweet ends.\n Please respond with ‘yes’ or ‘no’.	204/1536	F_1_-score
	E		News vs. opinions	Please determine if the provided tweet below is news or opinion.\n News: if the tweet report news or a fact.\n Opinion: if the tweet expresses a person’s opinion or thoughts.\n Tweet: “[INSERT DATA HERE]”. Now the tweet ends.\n Please respond with ‘News’ or ‘Opinion’.	597/1143	Micro F_1_
	F		Dialects	Please determine Whether the tweet is written in Modern Standard Arabic (MSA), North African dialect, or Middle Eastern dialect.\n Tweet: “[INSERT DATA HERE]”. Now the tweet ends.\n Please respond with MSA, North Africa, or Middle East.	898/209/596	Micro F_1_
	G		Blame and negative speech	Please determine if the provided tweet below contains blame, negative, or demoralizing speech. If so, respond “yes”. If not, respond “no”.\n Tweet: “[INSERT DATA HERE]”. Now the tweet ends.\n Please respond with ‘yes’ or ‘no’.	108/1625	F_1_-score
	H		Whether the tweet can be verified	Please determine if the provided tweet below contains information that can be verified and classified as Fake or Real. Note that this is NOT classifying Fake or Real. It is about determining if the tweet contains information that can be verified.\n Tweet: “[INSERT DATA HERE]”. Now the tweet ends.\n Please respond with ‘Is Not Verifiable’ or ‘Is Verifiable’.	775/449	Micro F_1_
	I		Worth fact-checking	Please determine if the provided tweet below contains an important claim or dangerous content that maybe be of worth for manual fact-checking.\n Tweet: “[INSERT DATA HERE]”. Now the tweet ends.\n Please respond with ‘Maybe’, ‘Yes’, or ‘No’.	360/229/205	F_1_-score
	J		Contain fake information	Please determine if the provided tweet below contains any fake information.\n Tweet: “[INSERT DATA HERE]”. Now the tweet ends.\n Please respond with ‘Maybe’, ‘Yes’, or ‘No’.	323/70/397	F_1_-score
ITED (Indonesian Twitter emotion detection)^45^		Indonesian data and an English prompt template	Classify (1) anger, (2) happy, (3) sadness, (4) fear, (5) love	\n This is a data labeling task. The task is emotion classification of a tweet.\n You need to find ONE emotion that best describe the provided tweet among the following 5 categories: anger, happy, sadness, fear, and love.\n Based on the content of the tweet, please choose the most appropriate category as your response.\n Tweet content: [INSERT DATA HERE]. Now the tweet ends.\n Please answer: Which one of the five emotion categories best does this tweet: anger, happy, sadness, fear, or love? Answer with one of them.	229/214/200/119/119	Micro F_1_
MAT (Misinformation on Arabic Twitter)^46^		Arabic data and an English prompt template	Classify (1) tweet that contained misinformation from (2) others	Please respond ‘yes’ if the provided tweet below contains misinformation. Otherwise, respond ‘no’.\n Tweet: “[INSERT DATA HERE]”. Now the tweet ends.\n Please respond with ‘yes’ or ‘no’.	189/1095	F_1_-score
WCV (Weibo COVID vaccine)^47^	A	Chinese data and Chinese-English code-mixing prompt templates	Weibo posts from personal accounts	Weibo post: [INSERT DATA HERE]. End of Weibo post. Please label whether this Weibo post is from a personal account about the COVID-19 vaccine. A post from a personal account could be expressing personal experiences, attitudes, thoughts, etc., as opposed to posts from governments, corporations, communities, or bots that do not contain personal opinions. Posts completely unrelated to the COVID-19 vaccine or simply reposting someone else’s post should also be excluded from this category. Answer yes or no.	568/349	F_1_-score
	B		Vaccine acceptance	Weibo post: [INSERT DATA HERE]. End of Weibo post. Please label whether this Weibo post mentions willingness to receive the COVID-19 vaccine. This refers to expressing support, acceptance, or willingness to get vaccinated. Answer yes or no.	323/245	F1-score
	C		Vaccine refusal	Weibo post: [INSERT DATA HERE]. End of Weibo post. Please label whether this Weibo post expresses unwillingness to receive the COVID-19 vaccine. Such posts typically express concerns about vaccination, skepticism, refusal, opposition, or lack of support for COVID-19 vaccination. Concerns about safety, effectiveness, etc., are also included in this category. Answer yes or no.	109/459	F_1_-score
	D		Vaccine is effective	Weibo post: [INSERT DATA HERE]. End of Weibo post. Please label whether this Weibo post mentions that the COVID-19 vaccine is effective. Effectiveness here refers to generating antibodies or having the effect of preventing COVID-19 (a positive evaluation of effectiveness). For example, statements like “can prevent infection” or “reduces severe cases and deaths” would qualify. Answer yes or no.	121/447	F_1_-score
	E		Vaccine is not effective	Weibo post: [INSERT DATA HERE]. End of Weibo post. Please label whether this Weibo post mentions that the COVID-19 vaccine is ineffective or has poor efficacy. Such posts may express doubts about the vaccine’s effectiveness, believe it to be ineffective, or suggest that mutations in the virus make it unable to generate antibodies or prevent COVID-19 (a negative evaluation of effectiveness). For example, statements like “cannot prevent infection,” “still got infected after vaccination,” or “the disease was still severe after vaccination” would qualify. Answer yes or no.	74/494	F_1_-score
	F		Vaccine is important	Weibo post: [INSERT DATA HERE]. End of Weibo post. Please label whether this Weibo post mentions that the COVID-19 vaccine is important. This refers to posts stating that the vaccine is important, necessary, essential, etc. Answer yes or no.	97/471	F_1_-score
	G		Risk perception	Weibo post: [INSERT DATA HERE]. End of Weibo post. Please label whether this Weibo post mentions a high-risk perception of the COVID-19 pandemic. This refers to posts that perceive the risk of the virus as high, the pandemic as very serious, or the threat to health as significant. Answer yes or no.	98/470	F_1_-score
	H		Negative information and misinformation	Weibo post: [INSERT DATA HERE]. End of Weibo post. Please label whether this Weibo post is about negative information regarding vaccines, such as vaccine rumors, anti-vaccine movements, anti-intellectual or anti-science movements, or negative vaccine-related incidents. Answer yes or no.	49/519	F_1_-score

The evaluation benchmark is based on manually annotated social media datasets. The source of the datasets, language, and the distribution of labels were presented. None of the evaluation datasets were used during the training of PH-LLM models. Note: The prompt templates for the WCV dataset were initially written in Chinese. Therefore, the original prompts were presented in our GitHub repository (https://github.com/luoyuanlab/PH-LLM), whereas English translation was shown in this table. We construct the prompt templates according to the original data annotation strategy described by the creator of the source datasets without any paraphrasing, whenever possible.

### Constructing Infoveillance Instructions (I^2^) Dataset

The I^2^ dataset was developed using 30 social media datasets included in the training corpus. Figure 3 illustrated the process of transforming previously annotated social media-based public health infoveillance datasets into instruction datasets, which was applied to create I^2^, an integrated instruction-tuning datasets for training PH-LLM. The datasets in I^2^ were sourced from a total of 30 manually annotated social media datasets from prior studies and were either monolingual or multilingual. Detailed information about each training dataset in I^2^ is provided in Supplementary Table 1. Typically, social media datasets collected from the Internet contain two primary entries: the social media post (or its ID), and corresponding annotation(s) (e.g., 0 and 1). For datasets containing only post IDs (all sourced from X, formally known as Twitter), we retrieved the actual textual content of the post (excluding replies) via the official X API^28^. Each post was transformed into an instruction comprehensible to humans, and its annotation(s) were converted into the gold-standard response for that instruction. Templates were applied to transform social media posts into instructions, as shown in Fig. 3.

To enhance the multilingual capabilities of PH-LLM models, templates for developing the I^2^ dataset were translated into 29 languages supported by Qwen 2.5. The list of supported languages is available in Supplementary Material 1.

The original social media posts were not translated. Instead, for each post in I^2^, a template in one of the 29 languages was randomly assigned. Each instruction was a combination of one social media post and one template^27^. Templates were initially crafted in Chinese or English, and subsequently translated into 28 additional languages using the web interface of ChatGPT-4o (https://chatgpt.com/).

### Constructing Public Health Question Answering (PHQA) Dataset

To construct the PHQA dataset, we employed a two-step process. First, we applied a keyword-based filtering approach to extract public health-related instruction-output pairs from three datasets: PubMed summarization, Meadow medical flashcards, and OpenOrca, as a supplement to the training set^29–31^. The keywords used for filtering are presented in Supplementary Material 1. Second, we selected subsets from the MedMCQA dataset, focusing specifically on two subjects: Social/Preventive Medicine and Psychiatry^32^. We further sampled 10,000 records from the MentalLLaMA collection, a question-answering dataset regarding mental health derived from gpt-3.5-turbo^13^. We also supplemented the PHQA with a subset of Bactrian-X^33^, a multilingual instruction-output dataset generated by gpt-3.5-turbo, to reinforce the multilingual capabilities of our instruction-tuned model.

Merging I^2^ and PHQA yielded our training set consisting of 593,100 instruction-output pairs (Supplement Table 1).

### Constructing evaluation datasets

For the evaluation benchmark, we selected 10 high-quality, manually annotated social media datasets (Table 5) that were distinct from the training datasets. We included tasks in these datasets where minority classes comprised at least 5% of the data, as extremely imbalanced tasks can cause metric fluctuations and may be less relevant to public health. As illustrated in Fig. 3, we transformed each record in the evaluation datasets into instruction-output pairs based on prompt templates. The original evaluation datasets, prior to the application of instruction templates, were in English, Chinese, Arabic, or Indonesian, whereas the prompt templates for the evaluation datasets were in English or Chinese-English code-mixing (Table 5). Putting datasets and prompt templates together yielded six evaluation datasets with 19 tasks in English and four multilingual evaluation datasets (two in Arabic-English code-mixing, one in Indonesian-English code-mixing, and one in Chinese-English code-mixing), encompassing 20 tasks. In total, we collected 52,158 instruction-output pairs from 39 tasks across 10 datasets in the evaluation benchmark. Detailed prompt templates for each evaluation task are shown in Table 5.

### Instruction-tuning of the PH-LLM Models

Qwen-2.5, a foundation model developed by Alibaba, was pretrained using up to 18 trillion tokens across more than 29 languages. The model family includes both base model (pretrained only), and instruction models (further trained through instruction-tuning and other methods)^24^. We chose the instruction-tuned version of Qwen2.5 as the backbone for the PH-LLM models, given its multilingual capabilities and superior performance in following human instructions^24^. Supporting over 29 languages, Qwen 2.5 enhances the potential applicability of PH-LLM in global health contexts, including low- and middle-income countries (LMICs).

To enable efficient LLM finetuning, we utilized quantized low-rank adaption (QLoRA)^34,35^. For instruction-tuning, we followed previous studies and trained our models over 3 epochs^15,36^, with an effective batch size of 256, a cut-off length of 1024 tokens, a learning rate of 0.00005, incorporating cosine annealing with a warm-up ratio of 0.1, and LoRAPlus learning rate ratio of 16. LoRAPlus builds upon standard LoRA by introducing separate upward and downward scaling coefficients, which makes more stable optimization and improved adaptation efficiency. The training process involved a total of 20 H100 GPUs at Northwestern University^37^. The instruction-tuning process also adhered to Qwen 2.5’s prompt format to maintain consistency. Elaborations on instruction tuning are available in Supplementary Material 1.

### Model evaluation

We evaluated the zero-shot performance of PH-LLM models—PH-LLM-0.5B, PH-LLM-1.5B, PH-LLM-3B, PH-LLM-7B, PH-LLM-14B, and PH-LLM-32B—against a wide array of open-source and proprietary LLMs, including GPT-4o (version 2024-05-13), Llama-3.1-72B-Instruct, Mistral-Large-Instruct-2407, and Qwen2.5-72B-Instruct^24–27^. During the evaluation of the open-source models (PH-LLM, Llama, Mistral, BLOOMZ, and Qwen 2.5), we used 4-bit quantization with QLoRA to enhance computational efficiency, using a total of 20 H100 GPUs at Northwestern University. GPT-4o (version 2024-05-13) and GPT-4o mini (version 2024-07-18) were deployed on the Microsoft Azure platform.

### Performance metrics

All evaluation datasets focused on a classification task, which represents the predominant type of annotated datasets in public health infoveillance. For classification tasks where only one category was relevant to public health, model performance was assessed using the F_1_-score. For tasks involving multiple categories of public health significance, we reported the micro F_1_-score to account for class imbalance. The formulas for calculating $\mathrm{precision}$, recall, F_1_-score, and micro F_1_-score are presented in Supplementary Material 1. Bootstrap sampling (*n* = 1000) was used to estimate the confidence intervals of model performance (see Supplementary Data).

## Supplementary information

Supplementary materials

Supplementary Data

## Data Availability

The PH-LLM models and code in this study are publicly available in our GitHub repository ([https://github.com/luoyuanlab/PH-LLM](https://github.com/luoyuanlab/PH-LLM)). This study utilized a publicly available repository called LLaMA-Factory ([https://github.com/hiyouga/LLaMA-Factory](https://github.com/hiyouga/LLaMA-Factory) for model training and evaluation. We also provide source links for each dataset used in the study. However, due to platform-specific policies of social media providers, we cannot share the raw data directly.
