# Supplementary material for: A suite of large language models for public health infoveillance
Source: NPJ Digit Med. 2026 Feb 23;9:270. doi: 10.1038/s41746-026-02435-6 (PMC13039744; doi:10.1038/s41746-026-02435-6)
Supplement: Supplementary file 1 — Supplementary materials [file 41746_2026_2435_MOESM1_ESM.pdf]

Supplementary Material 1

Supplementary Table 1. Instruction tuning datasets for model training

Data	Language	Source	Task	Topic	Size
I^2					
Vaccine attitudes					
WHV (Weibo HPV vaccine)[1]	Chinese	Weibo	Hierarchical classification	HPV vaccine	23,000
TCV (Twitter COVID-19 vaccine)[2]	English	Twitter (X)	Hierarchical classification	COVID-19 vaccine	53,000
Mental health					
WCE (Weibo COVID emotion)[3]	Chinese	Weibo	7-class classification	Sentiment analysis	10,500
SR (Stress – Reddit)[4]	English	Reddit	Binary classification	Stress detection	3,000
DR (Depression – Reddit)[5]	English	Reddit	4-class classification	Depression detection	500
PEH (perceived emotions in hurricane)[6]	English	Twitter (X)	Binary classification	Identify perceived emotions in hurricane	10,000
UEC (emotion classification in Urdu)[7]	Urdu	Twitter (X)	Multilabel classification	Emotion classification in Urdu	6,000
SemEval-2020 task 9[8]	Hindi-English	Twitter (X)	Binary classification	Sentiment analysis	12,000
TO (Twitter optimists)[9]	English	Twitter (X)	3-class classification	Classify “optimistic,” “pessimistic,” or “neutral” tweets	6,000
VT (vulgarity on Twitter)[10]	English	Twitter (X)	5-class classification	Sentiment analysis	2,500
Nonpharmacological interventions					
WCT (Weibo COVID test)[11]	Chinese	Weibo	Hierarchical	Public response to	115,000

			classification	COVID-19 test in China	
Hate speech					
IHS (Indonesian hate speech)[12]	Indonesian	Twitter (X)	Binary classification	Hate speech and abusive language detection	10,000
BHS (Bengali hate speech)[13]	Bengali	YouTube and Facebook	Binary classification	Detect hate speech on YouTube and Facebook about various topics	20,000
KHS (Korean hate speech)[14]	Korean	Naver (an entertainment news platform)	Binary classification and 3-class classification	Detect hate speech and gender bias in comments	8,500
ToLD-BR (toxic language dataset for Brazilian Portuguese)[15]	Portuguese	Twitter (X)	Binary classification	Detect homophobia, obscene, insult, racism, misogyny, and xenophobia	11,000
YAB (YouTube anti-social behavior)[16]	Arabic	YouTube	Binary classification	Detection of offensive language in Arabic YouTube comments	5,000
AD (aggression detection)[17]	Hindi-English	Facebook, Twitter (X)	3-class classification	Aggression detection in Hindi-English Code-Mixed social media	8,000
UTT (Urdu Threating Tweets)[7]	Urdu	Twitter (X)	Binary classification	Threatening Tweet in Urdu	4,000
HSTW (hate speech from Twitter and	English	Twitter (X), Whisper		Hate speech from Whisper	1,000

Whisper) [18]				and Twitter (X)	
HSOL (hate speech and offensive language)[19]	English	Twitter (X)	3-class classification	Hate Speech and Offensive Language	48,000
SemEval-2019 task 6 [20]	English	Twitter (X)	Hierarchical classification	Offensive language classification	7,900
SemEval-2020 task 12[21]	English	Twitter (X)	Hierarchical classification	OffensEval 2020 (multilingual offensive language detection)	3,000
SemEval-2023 task 10 (Subtask B)[22]	English	Gab and Reddit	4-class classification	Classification of offensive and aggressive sexism posts	2,000
Let-Mi (misogynistic language on Arabic Levantine Twitter)[23]	Arabic	Twitter (X)	Hierarchical classification	Classify misogynistic replies towards popular female journalists' tweets	5,500
RP (Rheinische Post)[24]	German	Rheinische Post (Newspaper)	Binary classification	Classify aggressive comments of news articles	3,000
SemEval-2016 task 6[25]	English	Twitter (X)		Abortion opinion analysis	600
SemEval-2023 task 10 (Subtask A)[22]	English	Gab and Reddit	Binary classification	Binary sexism detection	10,000
MLMA (multilingual and multi-aspect hate speech analysis)[26]	Arabic, French, and English	Twitter (X)	Multilabel and multiclass	Classify the hostility type and the target of the tweet	4,000
Misinformation					
AFN (Arabic	Arabic	Twitter (X)	Binary	Identify	1,500

fake news)[27]			classification	fake news	
FC (fact-checking for public health claims)[28]	English	Snopes, Politifact, TruthorFiction, FactCheck, FullFact, Associated Press, Reuters News, and Health News Review	QA	Public health claims' fact-checking	8,000
Public Health QA					
MedMCQA[29]	English	multiple-choice question answering (MCQA) dataset about real world medical entrance exam questions	QA	Filtered by subject name: "Social & Preventive Medicine" or "Psychiatry"	15,000
MentalLLaMA QA[30]	English	gpt-3.5	QA	Built with gpt-3.5, based on annotated mental health datasets from social media	10,000
PubMed summarization[31]	English	PubMed	QA	Generate title based on the abstract of a research paper. Filtered using public health keywords*	5,000
Meadow medical flashcards[32]	English	Anki medical curriculum flashcards	QA	Flashcards by medical students to assist learning. Filtered using public health keywords*	1,400
OpenOrca[33]	English	gpt-3.5, gpt-4	QA	A general instruction-tuning	140,000

				dataset. Filtered using public health keywords*	
Bactrian-X[34]	24 languages	gpt-3.5	QA	Generated using gpt-3.5, using prompts translated from Alpaca and Dolly	19,200

Supplementary Table 2. Potential applications of PH-LLM

Field	Potential application
Vaccine attitude	Detecting cross-country and subnational trends in vaccine acceptance, misinformation, and vaccine hesitancy, using the World Health Organization's "3Cs" (confidence, complacency, convenience) framework[2,35,36]
	Tracking vaccine beliefs and concerns on social media close to real-time (e.g., based on Health Belief Model)[1,37]
	Detecting correlations between vaccine attitudes and disinformation on social media and real-world vaccine coverage[2,38]
Mental health	Identifying at-risk individuals with mental health issues[39]
	Measuring the prevalence of mental health issues[24,40]
	Tracking mental health during public health emergencies[40]
Nonpharmacological interventions (NPIs)	Sentiment following NPIs[41]
	Attitudes towards NPIs [11]
Hate speech	The impact of temperature on prevalence of hate speech[42]
Misinformation	Identifying widespread HPV vaccine misinformation on Reddit[43]

We also cited relevant papers for readers interested in more detailed discussions of each public health application, noting that PH-LLM can be applied in place of the machine learning or manual content analysis methods used in those studies.

Supplementary Table 3. Few shot results of all LLMs in English datasets (2S: 2 shot)

	CAVES-A-2S	*CAVES-B-2S*	*CAVES-C-2S*	*CAVES-D-2S*	*CAVES-E-2S*	*CAVES-F-2S*	*CAVES-G-2S*
bloomz-7b1-mt	12	12.3	20.7	12.7	24.8	47.7	24.7
PH-LLM-0.5B	12.2	17.3	24.1	13.6	22.9	40.3	20.5
PH-LLM-1.5B	19.3	34.3	36.5	18.2	35.5	59.5	35.1
PH-LLM-3B	23.7	27.3	35.9	20.1	41.3	71.5	34.7
PH-LLM-7B	28.4	35.2	41.4	17.7	40.4	72.1	41.2
PH-LLM-14B	40.5	43.4	52	24.4	42	78.6	47.8
PH-LLM-32B	37.3	53	55.9	27.8	43.5	79.4	49.3
Llama-3.2-1B-Instruct	12.8	14.7	22.9	11.9	26	55.6	28.8
Llama-3.2-3B-Instruct	16.1	18.5	25.8	13	30.3	59.1	29.5
Llama-3.1-8B-Instruct	21.4	26	33.5	16.9	37.4	74.7	32.8
Llama-3.1-70B-Instruct	30.2	50.4	41.6	22.2	44.4	72.4	38.6
Mistral-Nemo-Instruct-2407	30.6	28.8	40.5	14.8	31.4	75.1	31.1
Mistral-Small-Instruct-2409	0	51.5	15.7	29.9	56.9	61.5	50
Mistral-Large-Instruct-2407	34	52.7	48.4	25.2	47.7	76.2	46.6
Qwen2.5-0.5B-Instruct	12.2	15.8	22.8	10.4	26.4	57	30.5
Qwen2.5-1.5B-Instruct	18.2	22.3	25.6	13.1	28.1	58.7	30.3
Qwen2.5-3B-Instruct	21	21.2	37.7	16.4	42.2	75.1	33.1
Qwen2.5-7B-Instruct	32.4	40.5	47.9	25.6	50.6	68.2	40.7
Qwen2.5-14B-Instruct	35.3	45.2	45.7	25.2	47.5	73.7	51.3
Qwen2.5-32B-Instruct	25.2	51.2	49.2	22.4	40	79.6	41.2
Qwen2.5-72B-Instruct	25.5	47.2	29.7	17.4	35.6	78.9	46.3
GPT-4o mini	25.6	51.9	42.8	22	33.8	74.2	34.7
GPT-4o	24.3	58.4	37.5	25.8	44.9	76.8	42.8

	CC-2S	*Ethos-2S*	*GHC-A-2S*	*GHC-B-2S*	*MC-A-2S*	*MC-B-2S*	*MC-C-2S*	*MC-D-2S*	*MC-E-2S*
bloomz-7b1-mt	48.2	38.3	15.9	13.4	30	29	20.8	20.4	12.7
PH-LLM-0.5B	55.4	60.7	26.9	20	38.5	30.7	21.3	22.4	8.2
PH-LLM-1.5B	81.2	73.9	37.8	41.8	37.7	48.9	33	18.9	4.1
PH-LLM-3B	84.2	81.5	42.4	42.8	47	38.9	36.1	20.7	18.2
PH-LLM-7B	91.5	80	41.8	39.9	46.8	67.2	47.7	28.3	21.3
PH-LLM-14B	93.2	81.7	48.4	46.2	44.6	83.7	49.7	39.9	36.2
PH-LLM-32B	93.5	86.7	47	43.5	49.7	64.7	55.6	32.3	25.7
Llama-3.2-1B-Instruct	62.8	59	14.1	0	35.8	28.6	21.6	14.7	12.2
Llama-3.2-3B-Instruct	90.6	9.1	27.3	17.6	42.9	36.4	28.6	22.7	15.8
Llama-3.1-8B-Instruct	76.7	13.2	36.1	23.3	44.8	70.6	43.7	36	20.1
Llama-3.1-70B-Instruct	90.5	85.7	45.3	34.9	63.3	77.5	55	61.8	25.4
Mistral-Nemo-Instruct-2407	90.6	84.6	49.3	32	50.4	66	47.8	34.1	9.8
Mistral-Small-Instruct-2409	90.6	79.5	39.4	51.1	54.9	72.8	66.7	53.9	10.4
Mistral-Large-Instruct-2407	93	88.1	48.7	43	49.9	75.8	69.1	58.2	25.3
Qwen2.5-0.5B-Instruct	30.1	6.5	16.6	10.9	15.3	21	15.9	12.2	0
Qwen2.5-1.5B-Instruct	90.3	70.7	24.9	27.6	5	36.6	27.1	0	2.7
Qwen2.5-3B-Instruct	82.2	77.5	30.8	36.1	48.4	52.2	40	25.3	17.6
Qwen2.5-7B-Instruct	87.1	77.8	46.7	46.4	49.8	70.2	58.3	34	21.4
Qwen2.5-14B-Instruct	89.8	87.5	47.4	46.7	58.1	75.5	51.2	42.1	20.8
Qwen2.5-32B-Instruct	90.9	87.5	45.2	39.6	54.8	76.3	55.1	49.4	22.4
Qwen2.5-72B-Instruct	91.5	85.4	42.5	40.3	57.1	78.7	56.8	44.1	26.4
GPT-4o mini	86.5	70.5	42.8	33.8	55.9	80.2	45.6	44.6	23.9
GPT-4o	94.7	78.5	45.1	30.3	56.7	79.2	62	54.7	21.3

	TCT-A-2S	*TCT-B-2S*	*TCT-C-2S*	***average***
bloomz-7b1-mt	51.3	59.5	22.8	**27.2**
PH-LLM-0.5B	68.9	40.9	26	**30.0**
PH-LLM-1.5B	42.4	18.5	8.1	**36.0**
PH-LLM-3B	29.4	24.6	69.7	**41.6**
PH-LLM-7B	76	50.2	0	**45.6**
PH-LLM-14B	68.5	44.4	74.6	**54.7**
PH-LLM-32B	65	47.5	71.9	**54.2**
Llama-3.2-1B-Instruct	37.9	59.6	28.6	**28.8**
Llama-3.2-3B-Instruct	62.6	44.4	30.3	**32.7**
Llama-3.1-8B-Instruct	20	42.5	42.9	**37.5**
Llama-3.1-70B-Instruct	78.9	35.1	72.5	**54.0**
Mistral-Nemo-Instruct-2407	58.7	5	36	**43.0**
Mistral-Small-Instruct-2409	90.8	19.3	62.8	**50.4**
Mistral-Large-Instruct-2407	84.2	0	79.4	**<u>55.0</u>**
Qwen2.5-0.5B-Instruct	15.3	58.5	29.6	**21.4**
Qwen2.5-1.5B-Instruct	29.6	53.9	25.7	**31.1**
Qwen2.5-3B-Instruct	89.4	2	25.6	**40.7**
Qwen2.5-7B-Instruct	87.8	10.3	43.1	**49.4**
Qwen2.5-14B-Instruct	87.3	13.7	64.2	**53.1**
Qwen2.5-32B-Instruct	55.1	22	67.8	**51.3**
Qwen2.5-72B-Instruct	89.2	17	64.2	**51.3**
GPT-4o mini	64.2	41.2	57.4	**49.0**
GPT-4o	40.3	38.9	78.3	**52.1**

Descriptions of datasets and tasks presented: CAVES: A dataset concerning COVID-19 vaccine (Classification task A: vaccine not necessary, B: freedom, C: companies making money, D: distrust in policymakers, E: clinical trials were not reliable, F: side effects, G: distrust in effectiveness); CC: a dataset classifying personal narrative and news; Ethos: classifying hate speech; GHC: a hate speech dataset (Classification task A: assaults on human diginity, B: offensive language towards individuals); MC: a COVID-19 misinformation dataset (Classification task A: calling out or correction, B: conspiracy, C: politics, D: sarcasm or satire, E: false fact or prevention); TCT: a dataset on COVID-19 test: (Classification task A: tweets sent by individual users about COVID-19 test, B: supporting mass COVID-19 testing, C: mentioning COVID-19 test for certain subpopulations).

Supplementary Table 4. Few shot results of all LLMs in multilingual datasets (2S: 2 shot)

	AHSFN-A-2S	*AHSFN-B-2S*	*AHSFN-C-2S*	*AHSFN-D-2S*	*AHSFN-E-2S*	*AHSFN-F-2S*	*AHSFN-G-2S*	*AHSFN-H-2S*	*AHSFN-I-2S*	*AHSFN-J-2S*
bloomz-7b1-mt	11.8	26.3	28.8	20.6	30.9	30.3	11.3	22.5	38.9	12.6
PH-LLM-0.5B	31.2	14.2	26.3	22.2	35	34.6	16.5	62.9	24	2.3
PH-LLM-1.5B	42.8	60.1	31.2	28.6	69.7	50.2	17.5	67.3	24.9	21.2
PH-LLM-3B	48.2	76.2	42.7	23.9	64.3	54.8	21.7	74.3	24.8	24.7
PH-LLM-7B	63.7	72.7	61	28.4	83.6	61.1	30.4	82.2	47.4	31.7
PH-LLM-14B	70.4	82.1	63.4	26.5	81.4	53.5	23.5	81	32	37
PH-LLM-32B	63.2	87.1	67.3	33.1	77.9	69.7	30.9	82.4	37	49.3
Llama-3.2-1B-Instruct	14.7	27.3	16.2	14.6	34.3	52.7	11.8	42.7	27.4	5
Llama-3.2-3B-Instruct	30.8	43.3	29.3	24.1	83.9	37.1	12.9	82.4	42.3	22
Llama-3.1-8B-Instruct	46.2	82.8	36.6	26.6	82.4	61.4	18.9	79.5	33	20.7
Llama-3.1-70B-Instruct	60.9	87.7	69.9	33.7	82.8	69.8	35.2	92.2	33.8	38.2
Mistral-Nemo-Instruct-2407	56	76	69.6	38.2	84	66.4	35.3	83.8	13.1	32.3
Mistral-Small-Instruct-2409	17.1	65.5	33.7	42.9	86.4	65	34	87	17.5	23.8
Mistral-Large-Instruct-2407	47.7	89.8	71.4	40.9	85.9	66.7	35.6	87.2	32.9	42
Qwen2.5-0.5B-Instruct	9	30.3	29	21.1	52.4	52	11.2	45.7	12.4	14
Qwen2.5-1.5B-Instruct	47	51.1	24	23.4	44.9	55.8	11.4	66.7	0	0
Qwen2.5-3B-Instruct	34.4	78.8	34.5	39.1	82.4	39.9	22.8	74.8	3.4	5.1
Qwen2.5-7B-Instruct	60	77.9	70.3	36.4	79.8	63.7	34.1	87.2	25.5	20
Qwen2.5-14B-Instruct	75.8	87.9	69.9	36.7	85.3	56.7	35.5	89.1	34.6	10.7
Qwen2.5-32B-Instruct	55.6	89.8	74.2	39.6	84	75.9	31.8	89.2	31.6	33
Qwen2.5-72B-Instruct	63.2	89.4	76.9	36.1	84	71.8	35.1	85.7	31.8	33.3
GPT-4o mini	50.5	85.8	65.4	33.6	84	63.6	29.8	83.2	33.5	40.8
GPT-4o	48.8	89.5	74	37.5	84	73.5	30.9	88.7	25.9	26.2

	ITED-2S	*MAT-2S*	*WCV-A-2S*	*WCV-B-2S*	*WCV-C-2S*	*WCV-D-2S*	*WCV-E-2S*	*WCV-F-2S*	*WCV-G-2S*	*WCV-H-2S*	***average***
bloomz-7b1-mt	17.7	24.2	53.4	56.8	24.8	29.8	19.1	23.6	25.7	18.6	**26.4**
PH-LLM-0.5B	28.4	22.1	58.4	49.6	32.3	36.2	24.8	25.3	30.8	29.8	**30.3**
PH-LLM-1.5B	44.6	24.1	31.1	51	40.4	40	53.7	37.9	50	28.3	**40.7**
PH-LLM-3B	45.7	30.9	48.4	66.7	46.9	46.4	50.7	35.2	47.8	27.5	**45.1**
PH-LLM-7B	67.9	46	86.7	82	33.1	60.3	68.7	63.3	58.6	40.7	**58.5**
PH-LLM-14B	72.5	49.4	77.3	81.1	55.5	59.3	71.5	51.9	60.5	35	**58.2**
PH-LLM-32B	75.3	57.4	83.5	80.8	57.9	68.3	65.9	53.2	62	45.8	**62.4**
Llama-3.2-1B-Instruct	24.4	7.6	2.1	68.5	12.4	23.3	17.3	24.2	14.4	17.9	**22.9**
Llama-3.2-3B-Instruct	60.6	26.3	65.8	52	27.2	33.1	27.5	30.5	34.4	14.4	**39.0**
Llama-3.1-8B-Instruct	64.9	32.4	76.7	10.5	21.3	53.2	34.3	31.5	41.9	31.1	**44.3**
Llama-3.1-70B-Instruct	76.5	48.2	77.2	79.7	55.7	55.4	53.3	46.5	56.9	37.5	**59.6**
Mistral-Nemo-Instruct-2407	68.9	40.2	65.8	35.2	54.2	47.1	30.9	37.1	48.6	46.2	**51.4**
Mistral-Small-Instruct-2409	71.9	1.1	29.5	64.9	58	48	15	43.8	56.4	41	**45.1**
Mistral-Large-Instruct-2407	75.4	48.9	31	28.9	65	38.7	54.8	41.9	59.6	44.9	**54.5**
Qwen2.5-0.5B-Instruct	25.4	21.3	76.5	73	31.3	37	22.1	29.5	29.3	16.1	**31.9**
Qwen2.5-1.5B-Instruct	54.8	9.5	57.8	59	16	44.6	16.8	25.3	37.8	10.3	**32.8**
Qwen2.5-3B-Instruct	57.8	29.4	42.6	40.3	23.8	48.5	34.1	34.4	39.9	29.1	**39.8**
Qwen2.5-7B-Instruct	73	39	73.5	47	54.1	52.7	50.5	32.4	45.8	42.7	**53.3**
Qwen2.5-14B-Instruct	71.5	47.1	72.4	32.1	60.7	59.6	59.1	48.7	51.2	40.6	**56.3**
Qwen2.5-32B-Instruct	74.2	51.7	71.8	36	63.6	57.4	56.3	37.6	54.4	38.7	**57.3**
Qwen2.5-72B-Instruct	77.2	51	53	28.2	63.2	60.3	60.4	41.5	61.1	52.2	**57.8**
GPT-4o mini	77.9	45.4	70.8	57.9	58.6	55.1	61.4	47.3	57	37.4	**57.0**
GPT-4o	75.1	48.3	66.4	63.4	65.1	65.4	61.4	58.4	58.5	42	**59.2**

Descriptions of datasets and tasks presented: AHSFN: an Arabic dataset regarding hate speech and misinformation regarding COVID-19 (Classification tasks A: hate speech, B: cure or vaccine mentions, C: advice, D: encouraging tweets, E: news vs. opinions, F: dialects, G: blame and negative speech, H: whether the tweet can be verified, I: worth fact-checking, J: contain fake information); ITED: an Indonesian emotion detection dataset (classifying (1) anger, (2) happy, (3) sadness, (4) fear, (5) love); MAT: an Arabic dataset regarding classifying misinformation; WCV: a Chinese dataset regarding COVID-19 vaccine sentiment (Classification task A: classifying Weibo posts from personal accounts, B: vaccine acceptance, C: vaccine refusal, D: vaccine is effective, E: vaccine is not effective, F: vaccine is important, G: risk perception, H: negative information and misinformation).

Supplementary Figure 1. Training loss of PH-LLM

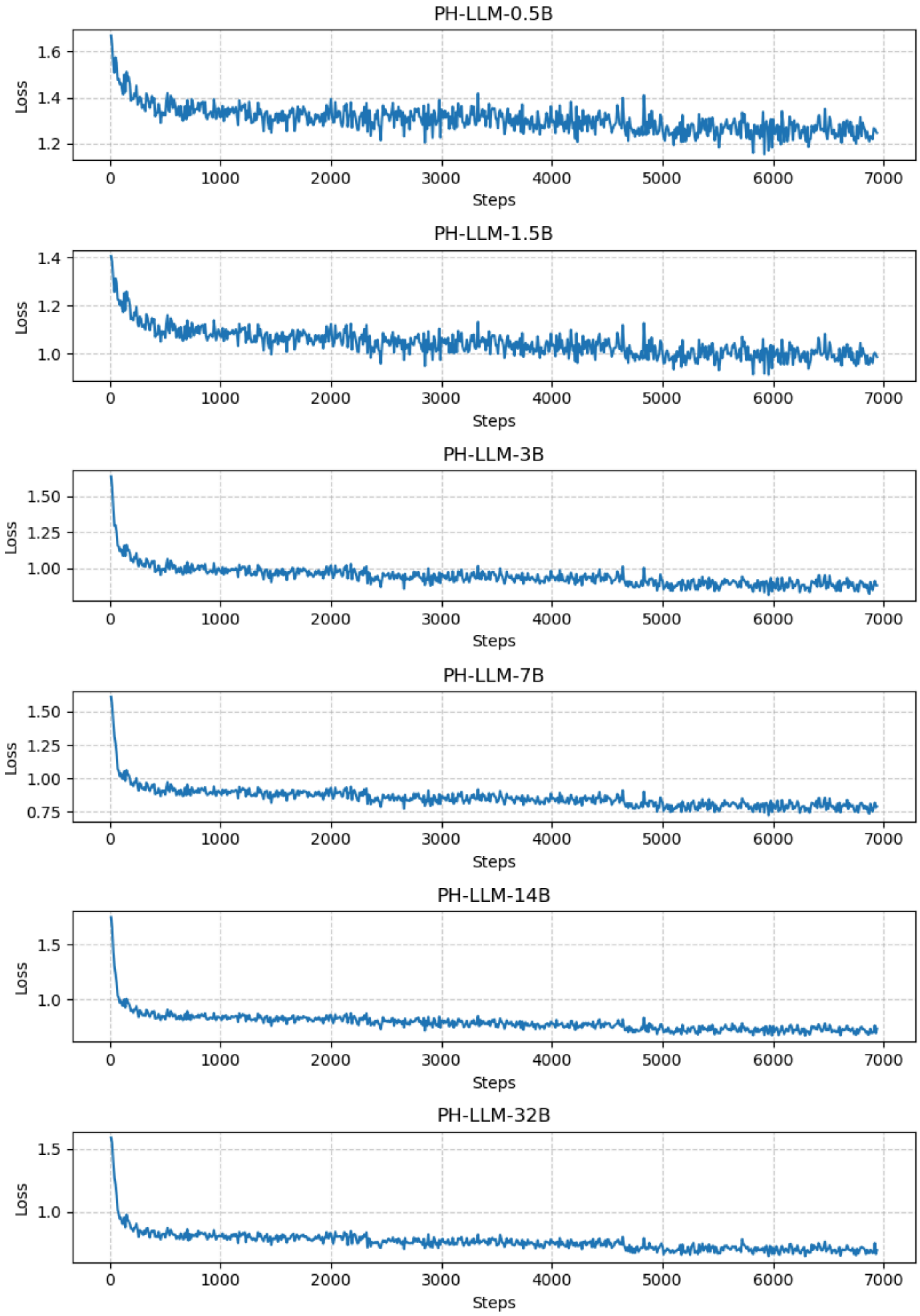

Supplementary Figure 2. Comparison of zero-shot performance on multilingual datasets between PH-LLM models and other open-source LLMs

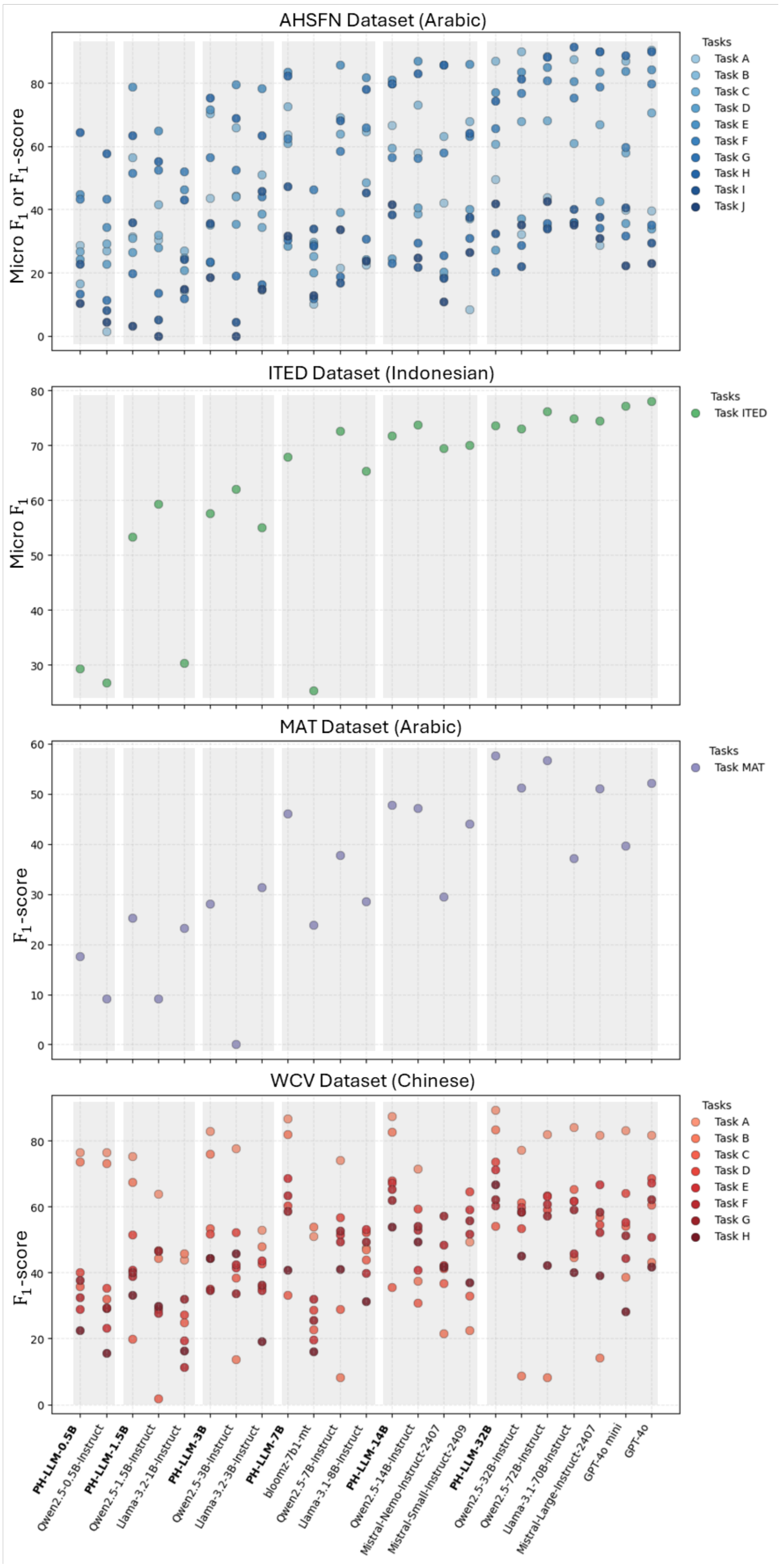

Statistical significant testing based on confidence intervals calculated using bootstrap sampling

For each task t and model m, let $\hat{\theta}_{t,m}$denote the estimated performance metric (higher is better), reported together with a two-sided 95% confidence interval

$$CI_{t,m} = [L_{t,m}, U_{t,m}], \tag{1}$$

the standard error is approximated as

$$SE_{t,m} = \frac{U_{t,m} - L_{t,m}}{2z_{0.975}}, \tag{2}$$

where $z_{0.975} = 1.96$.

For a given task t, we compare a PH-LLM model $p$against another model q.

Difference in estimates

$$\Delta_{t,p,q} = \hat{\theta}_{t,p} - \hat{\theta}_{t,q}, \hat{\theta}_{t,m} = \frac{L_{t,m} + U_{t,m}}{2}. \tag{3}$$

Assuming independence between model estimates,

$$SE_{t,p,q} = \sqrt{SE_{t,p}^2 + SE_{t,q}^2}. \tag{4}$$

Wald z-statistic

$$z_{t,p,q} = \frac{\Delta_{t,p,q}}{SE_{t,p,q}}. \tag{5}$$

We perform a one-sided hypothesis test to assess whether the PH-LLM model significantly outperforms the comparison model:

$$H_0: \hat{\theta}_{t,p} \le \hat{\theta}_{t,q}, H_1: \hat{\theta}_{t,p} > \hat{\theta}_{t,q}. \tag{6}$$

The corresponding one-sided p-value is computed as

$$p_{t,p,q} = 1 - \Phi(z_{t,p,q}), \tag{7}$$

where $\Phi(\cdot)$ denotes the cumulative distribution function of the standard normal distribution.

A comparison is considered statistically significant if

$$p_{t,p,q} < \alpha, \tag{8}$$

with $\alpha = 0.05$.

We presented all $p_{t,p,q}$ and $z_{t,p,q}$ in the ***Supplementary Data*** file.

Supplementary Figure 3. Win rate of each PH-LLM model against all baseline LLMs

For each task t and PH-LLM model p, we define the proportion of significant wins as

$$\mathrm{WinRate}_{t,p} = \frac{\sum_{q \in \mathcal{M}} \mathbb{I}\left(p_{t,p,q} < \alpha\right)}{\mid \mathcal{M} \mid}, \tag{9}$$

where $\mathcal{M}$ denotes the set of all baseline models (i.e., Llama, Mistral, Qwen, Bloomz, and GPT models) and $\mathbb{I}(\cdot)$ is the indicator function. $\mathrm{WinRate}_{t,p}$ equals 1 if the PH-LLM model significantly outperforms all other models on task t, and 0 otherwise.

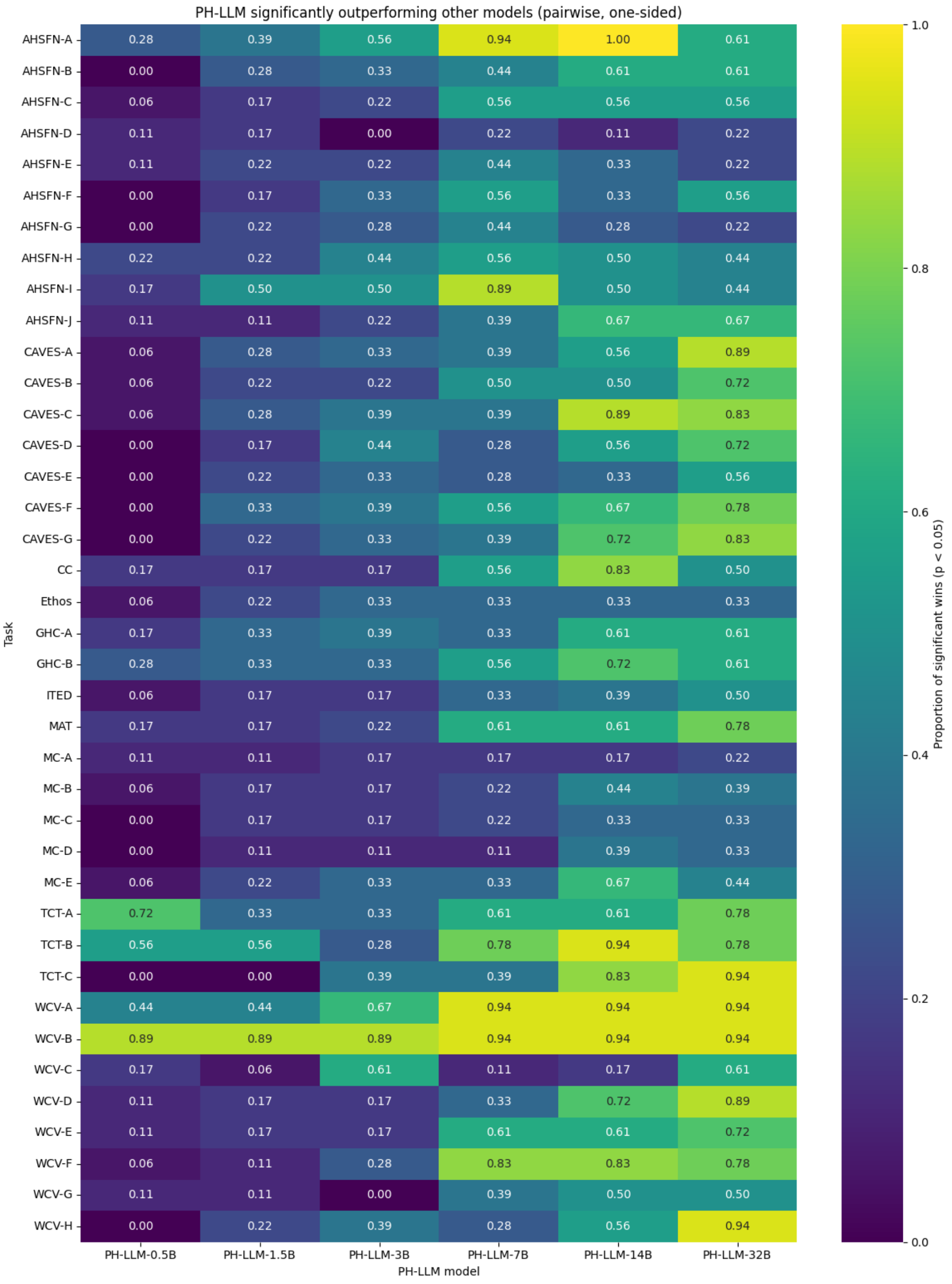

Public health keywords for filtering the datasets

public health, global health, health promotion, disease prevention, health education, health policy, health equity, health disparities, access to healthcare, health systems, community health, infectious diseases, chronic diseases, mental health, pandemic, epidemic, outbreak, obesity, diabetes, heart disease, cancer, hiv, aids, tuberculosis, malaria, smoking cessation, alcohol use, substance abuse, healthy eating, physical activity, exercise, nutrition, stress management, sleep hygiene, depression, anxiety, mental health awareness, suicide prevention, mental health services, reducing stigma, air pollution, water quality, sanitation, environmental hazards, climate change, environmental health, vaccine hesitancy, vaccine confidence, vaccination, immunization, herd immunity, vaccine uptake, immunization programs, vaccine safety, health literacy, health communication, misinformation, health campaigns, health promotion strategies, risk communication, digital health, telehealth, e-health, mhealth, health apps, health informatics, health data privacy, wearable technology, universal health coverage, healthcare reform, health insurance, healthcare access, health economics, health services research, healthcare quality, disaster preparedness, emergency response, public health emergencies, pandemic preparedness, contact tracing, maternal health, child health, infant mortality, reproductive health, family planning, prenatal care, breastfeeding, socioeconomic status, education, housing, food security, employment, social support, social determinants of health, health inequality, minority health, indigenous health, health justice, health advocacy, workplace safety, occupational hazards, occupational health, occupational stress, global burden of disease, health in developing countries, international health, global health initiatives, one health, behavior change, health behaviors, behavioral interventions, health belief model

Languages officially supported by Qwen 2.5

Arabic, Bengali, Burmese, Cebuano, Chinese, Czech, Dutch, English, French, German, Hebrew, Hindi, Indonesian, Italian, Japanese, Khmer, Korean, Lao, Malay, Persian, Polish, Portuguese, Russian, Spanish, Tagalog, Thai, Turkish, Urdu, Vietnamese

Instruction-tuning, LoRA, QLoRA, and LoRAPlus

Instruction-tuning is a technique of training large language models (LLMs) to follow natural language instruction. With instruction-output pairs covering diverse scenarios, LLMs were trained to follow human instruction, even on tasks that are unseen during model training. It has been widely applied to train LLMs in the healthcare domain[44,45].

Low-Rank Adaptation (LoRA)[46] introduces small, trainable low-rank matrices ΔW to the model's weight matrices W_o. Instead of updating the full weight matrix W_o, LoRA decomposes the update as: $\Delta W = BA$,where $A \in R^{r\times k}, B \in R^{d\times r}$and $\mathrm{r} \ll \min(\mathrm{d}, \mathrm{k})$, and update the original weight matrices W_o to

$$W_o = W_o + \Delta W \tag{10}$$

where, r is the rank, significantly smaller than d, k, reducing the number of trainable parameters. During training, $W_o$is frozen and does not receive gradient updates, while A and B contain trainable parameters updated with same learning rate. Note both W_o and $\Delta W = BA$, are multiplied with the same input, and their respective output vectors are summed coordinate-wise.

Building on this, Quantized Low-Rank Adaptation (QLoRA)[47] combines quantization with LoRA. The base model weights W are quantized to W_q using 4-bit NormalFloat precision to reduce memory usage. Meanwhile, to make sure the model performance is preserved, for the low-rank updates ΔW , it is stored with higher precision using 16-bit BrainFloat.

Low-Rank Adaptation plus (LoRAPlus)[48] is based on LoRA with separate learning rate setting for matrix A and B. For the standard LoRA proposed by Hu et al., the learning rate for A and B is the same. According to Hayou et al, such a setting provably leads to suboptimal learning when embedding dimension is large. Thus, in LoRAPlus, the learning rate of B is set to be $\lambda \times$ that of A, where $\lambda \gg 1$ is fixed.

We employ these methods together, which allow efficient fine-tuning of large language models and significantly reducing resource requirements compared to traditional approaches while maintaining performance.

Evaluation metrics: Precision, recall, $\mathbf{F_1}$ − score, and micro $\mathbf{F_1}$ − score

$$\text{precision} = \frac{\text{TP}}{\text{TP} + \text{FP}} \tag{11}$$

Where TP is the number of true positives (correctly predicted positive instances), andFP is the number of false positives (incorrectly predicted positive instances).

$$\text{recall} = \frac{\text{TP}}{\text{TP} + \text{FN}} \tag{12}$$

Where FN is the number of false negatives (relevant instances that were not retrieved)

Then the $\text{F}_1 - \text{score}$ is defined as

$$\text{F}_1 - \text{score} = 2 \times \frac{\text{Precision} \times \text{Recall}}{\text{Precision} + \text{Recall}} \tag{13}$$

This is calculated by focusing on TP, FP, and FN of a specific category, whereas

$$\text{micro F}_1 - \text{score} = 2 \times \frac{\text{Micro-Precision} \times \text{Micro-Recall}}{\text{Micro-Precision} + \text{Micro-Recall}} \tag{14}$$

where Micro-Precision and Micro-Recall are calculated using TP, FP, and FN aggregated across all classes.

Other Supplementary Material

Supplementary Data: 95% confidence intervals of model performance based on bootstrap sampling (n = 1,000), available in a separate worksheet file.

References

1 Wang, Y. *et al.* Trajectories of and spatial variations in HPV vaccine discussions on Weibo, 2018-2023: a deep learning analysis. *medRxiv*, 2023.2012. 2007.23299667 (2023).

2 Zhou, X. *et al.* Spatiotemporal trends in COVID-19 vaccine sentiments on a social media platform and correlations with reported vaccine coverage. *Bulletin of the World Health Organization* **102**, 32 (2024).

3 Lyu, X., Chen, Z., Wu, D. & Wang, W. in *Natural Language Processing and Chinese Computing: 9th CCF International Conference, NLPCC 2020, Zhengzhou, China, October 14–18, 2020, Proceedings, Part I 9.* 710-721 (Springer).

4 Turcan, E. & McKeown, K. Dreaddit: A reddit dataset for stress analysis in social media. *arXiv preprint arXiv:1911.00133* (2019).

5 Naseem, U., Dunn, A. G., Kim, J. & Khushi, M. in *Proceedings of the ACM Web Conference 2022.* 2563-2572.

6 Desai, S., Caragea, C. & Li, J. J. Detecting perceived emotions in hurricane disasters. *arXiv preprint arXiv:2004.14299* (2020).

7 Butt, S. *et al.* in *Proceedings of the 14th Annual Meeting of the Forum for Information Retrieval Evaluation.* 1-3.

8 Patwa, P. *et al.* Semeval-2020 task 9: Overview of sentiment analysis of code-mixed tweets. *arXiv preprint arXiv:2008.04277* (2020).

9 Ruan, X., Wilson, S. & Mihalcea, R. in *54th Annual Meeting of the Association for Computational Linguistics.* 320-325 (Association for Computational Linguistics).

10 Cachola, I., Holgate, E., Preoţiuc-Pietro, D. & Li, J. J. in *Proceedings of the 27th International Conference on Computational Linguistics.* 2927-2938.

11 Lin, L. *et al.* Public attitudes and factors of COVID-19 testing hesitancy in the United Kingdom and China: comparative infodemiology study. *JMIR infodemiology* **1**, e26895 (2021).

12 Ibrohim, M. O. & Budi, I. in *Proceedings of the third workshop on abusive language online.* 46-57.

13 Romim, N., Ahmed, M., Talukder, H. & Saiful Islam, M. in *Proceedings of International Joint Conference on Advances in Computational Intelligence: IJCACI 2020.* 457-468 (Springer).

14 Moon, J., Cho, W. I. & Lee, J. BEEP! Korean corpus of online news comments for toxic speech detection. *arXiv preprint arXiv:2005.12503* (2020).

15 Leite, J. A., Silva, D. F., Bontcheva, K. & Scarton, C. Toxic language detection in social media for Brazilian Portuguese: New dataset and multilingual analysis. *arXiv preprint arXiv:2010.04543* (2020).

16 Alakrot, A., Murray, L. & Nikolov, N. S. Dataset construction for the detection of anti-social behaviour in online communication in Arabic. *Procedia Computer Science* **142**, 174-181 (2018).

17 Kumar, R., Reganti, A. N., Bhatia, A. & Maheshwari, T. Aggression-annotated corpus of hindi-english code-mixed data. *arXiv preprint arXiv:1803.09402* (2018).

18 Mondal, M., Silva, L. A. & Benevenuto, F. in *Proceedings of the 28th ACM conference on hypertext and social media.* 85-94.

19 Davidson, T., Warmsley, D., Macy, M. & Weber, I. in *Proceedings of the international AAAI conference on web and social media.* 512-515.

20 Zampieri, M. *et al.* Semeval-2019 task 6: Identifying and categorizing offensive language in social media (offenseval). *arXiv preprint arXiv:1903.08983* (2019).

21 Zampieri, M. *et al.* SemEval-2020 task 12: Multilingual offensive language identification in social media (OffensEval 2020). *arXiv preprint arXiv:2006.07235* (2020).

22 Kirk, H. R., Yin, W., Vidgen, B. & Röttger, P. Semeval-2023 task 10: Explainable detection of online sexism. *arXiv preprint arXiv:2303.04222* (2023).

23 Mulki, H. & Ghanem, B. Let-mi: an arabic levantine twitter dataset for misogynistic

language. *arXiv preprint arXiv:2103.10195* (2021).
24 Assenmacher, D. *et al.* in *Thirty-fifth conference on neural information processing systems datasets and benchmarks track (Round 2).*
25 Mohammad, S., Kiritchenko, S., Sobhani, P., Zhu, X. & Cherry, C. in *Proceedings of the 10th international workshop on semantic evaluation (SemEval-2016).* 31-41.
26 Ousidhoum, N., Lin, Z., Zhang, H., Song, Y. & Yeung, D.-Y. Multilingual and multi-aspect hate speech analysis. *arXiv preprint arXiv:1908.11049* (2019).
27 Mahlous, A. R. & Al-Laith, A. Fake news detection in Arabic tweets during the COVID-19 pandemic. *International Journal of Advanced Computer Science and Applications* **12**, 778-788 (2021).
28 Kotonya, N. & Toni, F. Explainable automated fact-checking for public health claims. *arXiv preprint arXiv:2010.09926* (2020).
29 Pal, A., Umapathi, L. K. & Sankarasubbu, M. in *Conference on health, inference, and learning.* 248-260 (PMLR).
30 Yang, K. *et al.* in *Proceedings of the ACM on Web Conference 2024.* 4489-4500.
31 White, J. PubMed 2.0. *Medical reference services quarterly* **39**, 382-387 (2020).
32 Han, T. *et al.* MedAlpaca--an open-source collection of medical conversational AI models and training data. *arXiv preprint arXiv:2304.08247* (2023).
33 Mukherjee, S. *et al.* Orca: Progressive learning from complex explanation traces of gpt-4. *arXiv preprint arXiv:2306.02707* (2023).
34 Li, H., Koto, F., Wu, M., Aji, A. F. & Baldwin, T. Bactrian-x: Multilingual replicable instruction-following models with low-rank adaptation. *arXiv preprint arXiv:2305.15011* (2023).
35 Hou, Z. *et al.* Assessing COVID-19 vaccine hesitancy, confidence, and public engagement: a global social listening study. *Journal of medical Internet research* **23**, e27632 (2021).
36 Larson, H. J. *et al.* Measuring vaccine confidence: analysis of data obtained by a media surveillance system used to analyse public concerns about vaccines. *The Lancet infectious diseases* **13**, 606-613 (2013).
37 Du, J. *et al.* Leveraging deep learning to understand health beliefs about the Human Papillomavirus Vaccine from social media. *NPJ Digital Medicine* **2**, 27 (2019).
38 Wilson, S. L. & Wiysonge, C. Social media and vaccine hesitancy. *BMJ global health* **5** (2020).
39 Eichstaedt, J. C. *et al.* Facebook language predicts depression in medical records. *Proceedings of the National Academy of Sciences* **115**, 11203-11208 (2018).
40 Karmegam, D. & Mappillairaju, B. Spatio-temporal distribution of negative emotions on Twitter during floods in Chennai, India, in 2015: A post hoc analysis. *International journal of health geographics* **19**, 19 (2020).
41 Wang, J. *et al.* Global evidence of expressed sentiment alterations during the COVID-19 pandemic. *Nature Human Behaviour* **6**, 349-358 (2022).
42 Stechemesser, A., Levermann, A. & Wenz, L. Temperature impacts on hate speech online: evidence from 4 billion geolocated tweets from the USA. *The Lancet Planetary Health* **6**, e714-e725 (2022).
43 Du, J. *et al.* Using machine learning–based approaches for the detection and classification of human papillomavirus vaccine misinformation: Infodemiology study of reddit discussions. *Journal of Medical Internet Research* **23**, e26478 (2021).
44 Xie, Q. *et al.* Me llama: Foundation large language models for medical applications. *arXiv preprint arXiv:2402.12749* (2024).
45 Wu, C. *et al.* PMC-LLaMA: toward building open-source language models for medicine. *Journal of the American Medical Informatics Association*, ocae045 (2024).
46 Hu, E. J. *et al.* Lora: Low-rank adaptation of large language models. *arXiv preprint arXiv:2106.09685* (2021).
47 Dettmers, T., Pagnoni, A., Holtzman, A. & Zettlemoyer, L. Qlora: Efficient finetuning of quantized llms. *Advances in Neural Information Processing Systems* **36** (2024).
48 Hayou, S., Ghosh, N. & Yu, B. Lora+: Efficient low rank adaptation of large models.

arXiv preprint arXiv:2402.12354 (2024).
